# Grassland species differentially regulate proline concentrations under future climate conditions: an integrated biochemical and modelling approach

**DOI:** 10.1111/nph.13481

**Published:** 2015-06-02

**Authors:** Hamada AbdElgawad, Dirk De Vos, Gaurav Zinta, Malgorzata A. Domagalska, Gerrit T. S. Beemster, Han Asard

**Affiliations:** ^1^ Laboratory for Molecular Plant Physiology and BiotechnologyDepartment of BiologyUniversity of AntwerpB‐2020AntwerpBelgium; ^2^ Department of BotanyFaculty of ScienceUniversity of Beni‐SueifBeni‐Sueif62511Egypt; ^3^ Department of Mathematics and Computer ScienceUniversity of AntwerpB‐2020AntwerpBelgium; ^4^ Molecular Parasitology UnitDepartment of Medical SciencesInstitute of Tropical MedicineAntwerpBelgium

**Keywords:** drought, elevated CO_2_, elevated temperature, grassland species, metabolic control analysis, proline (Pro) metabolism

## Abstract

- Proline (Pro) is a versatile metabolite playing a role in the protection of plants against environmental stresses. To gain a deeper understanding of the regulation of Pro metabolism under predicted future climate conditions, including drought stress, elevated temperature and CO_2_, we combined measurements in contrasting grassland species (two grasses and two legumes) at multiple organisational levels, that is, metabolite concentrations, enzyme activities and gene expression.
- Drought stress (D) activates Pro biosynthesis and represses its catabolism, and elevated temperature (DT) further elevated its content. Elevated CO_2_ attenuated the DT effect on Pro accumulation.
- Computational pathway control analysis allowed a mechanistic understanding of the regulatory changes in Pro metabolism. This analysis indicates that the experimentally observed coregulation of multiple enzymes is more effective in modulating Pro concentrations than regulation of a single step. Pyrroline‐5‐carboxylate synthetase (P5CS) and pyrroline‐5‐carboxylate reductase (P5CR) play a central role in grasses (*Lolium perenne*,* Poa pratensis*), and arginase (ARG), ornithine aminotransferase (OAT) and P5CR play a central role in legumes (*Medicago lupulina*,* Lotus corniculatus*).
- Different strategies in the regulation of Pro concentrations under stress conditions were observed. In grasses the glutamate pathway is activated predominantly, and in the legumes the ornithine pathway, possibly related to differences in N‐nutritional status.

## Introduction

Proline (Pro) is an essential proteinogenic amino acid, and is also known as a stress defence molecule. It is a compatible solute that adjusts cellular osmotic potential, protects membranes and proteins, stabilizes photosystem II and protects plants against oxidative damage (Szabados & Savouré, 2010). Pro metabolism has a regulatory function in cell homeostasis and survival (Phang, 1985; Liang *et al*., 2013). An increased rate of Pro biosynthesis can help to maintain higher NADP^+^: NADPH ratios and stabilize the redox balance (Hare & Cress, 1997; Szabados & Savouré, 2010). Other proposed functions of Pro include storage and transfer of energy (Abraham *et al*., 2003; Szabados & Savouré, 2010; Verslues & Sharma, 2010). Taken together, it is a remarkably multifunctional molecule involved in plant stress defence.

The earth's current, anthropogenic climate change is manifested by increases in atmospheric CO_2_ and temperature and more frequent heat waves and drought spells (IPCC, 2012). It is becoming increasingly clear that elevated CO_2_ may modify the response of plants to environmental stresses, in particular by reducing stress impact and altering plant metabolism (Ainsworth & Rogers, 2007; Geissler *et al*., 2009; Li *et al*., 2014; Naudts *et al*., 2014; Zinta *et al*., 2014; Pandey *et al*., 2015). However, the molecular mechanisms underlying these effects are still largely unclear (Feng *et al*., 2014). In previous work we showed that elevated CO_2_ alleviated photosynthesis inhibition under drought stress conditions, and reduced stress impact through reducing photorespiration and formation of hydrogen peroxide (H_2_O_2_; AbdElgawad *et al*., 2015).

**Figure 1:**
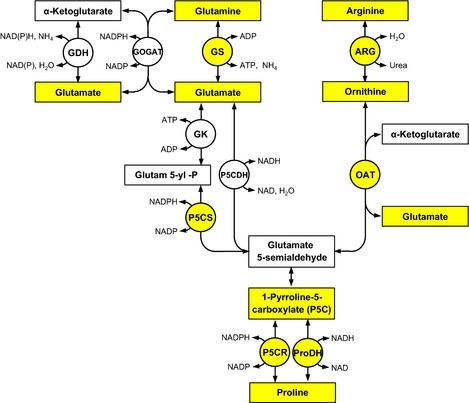
General overview of proline (Pro) metabolism. GDH, NADH‐glutamate dehydrogenase; GOGAT, glutamate synthase; GS, glutamine synthetase; GK, glutamate 5‐kinase; P5C, Δ1‐pyrroline‐5‐carboxylate; P5CS, P5C synthase; P5CDH, pyrroline‐5‐carboxylate dehydrogenase; P5CR, P5C reductase; PRoDH, proline dehydrogenase; ARG, arginase; OAT, ornithine‐d‐aminotransferase. The interconversion between P5C and glutamate‐5‐semialdehyde (GSA) is spontaneous. Highlighted are parameters quantitatively measured in this study.

Given the importance of Pro in plant stress, understanding the regulatory mechanisms of Pro accumulation is fundamental to our understanding of plant responses to global changes, and potentially useful in making crops stress‐tolerant. The glutamate (Glu) and ornithine (Orn) pathways can independently feed Pro synthesis (Fig. 1), and both can play a role in Pro accumulation during stress conditions (Hu *et al*., 1992; Roosens *et al*., 1999). In the Glu pathway, glutamine (Gln) is converted to Glu by glutamine synthetase (GS), reduced to glutamate‐5‐semialdehyde (GSA) by pyrroline‐5‐carboxylate synthetase (P5CS), which spontaneously converts to pyrroline‐5‐carboxylate (P5C) (Hu *et al*., 1992; Savoure *et al*., 1995). P5C is reduced to Pro by pyrroline‐5‐carboxylate reductase (P5CR) (Szoke *et al*., 1992; Verbruggen *et al*., 1993), a common enzyme in both pathways. In most plant species, P5CS is encoded by two genes (*P5CS1* and *P5CS2*), whereas P5CR is encoded by a single isoform (Verbruggen *et al*., 1993; Armengaud *et al*., 2004). Yoshiba *et al*. (1997); Huang *et al*. (2013), suggested that the P5CS‐mediated reaction is a rate‐controlling step in Pro synthesis and consistently it was reported as the predominant enzyme leading to Pro accumulation in drought‐stressed plants (Kim & Nam, 2013). P5CR is an important enzyme for Pro synthesis and has a critical role in cycling Pro and P5C between cellular compartments (Phang, 1985; Miller *et al*., 2009). P5C produced in the mitochondria, can be transported into the cytosol and be re‐reduced to Pro by cytosolic P5CR (Szabados & Savouré, 2010).

As an alternative pathway, Pro can be synthesized from Orn; which is generated from arginine by arginase (ARG), and transaminated to P5C by ornithine‐d‐aminotransferase (OAT) (Roosens *et al*., 1999; Yang *et al*., 2009). OAT is considered to control this pathway (Verbruggen & Hermans, 2008; Szabados & Savouré, 2010; Huang *et al*., 2013). Several studies have demonstrated a role of OAT in stress tolerance, and in regulating the plant cell redox homeostasis through modulating Pro metabolism (Delauney *et al*., 1993; Yamada *et al*., 2005).

Regulation of Pro content is also controlled by Pro catabolism. This occurs in mitochondria where Pro dehydrogenase (ProDH) generates P5C, which is converted by pyrroline‐5‐carboxylate dehydrogenase (P5CDH) to Glu. P5CS and OAT, ProDH is suppressed under stressful conditions, preventing Pro degradation (Kiyosue *et al*., 1996; Verbruggen *et al*., 1996). On the other hand, ProDH is induced upon recovery from stress (Kiyosue *et al*., 1996) or under normal growth conditions by applying high concentrations of Pro (Kishor *et al*., 2005).

In order to identify the reactions controlling Pro concentrations, and to clarify its physiological role, several studies have analyzed the effect of modifications in the Pro pathway in transgenic plants. For example, overexpression of P5CS or P5CR increased Pro content, which in turn stimulated plant growth under stress (Kishor *et al*., 1995; Hong *et al*., 2000; De Ronde *et al*., 2004; Hmida‐Sayari *et al*., 2005). Higher Pro content and elevated stress tolerance were also achieved by antisense inhibition of ProDH transcription (Mani *et al*., 2002; Nanjo *et al*., 2003) and overexpression of OAT genes (Roosens *et al*., 2002; Kishor *et al*., 2005; Wu *et al*., 2005). Although genetic manipulations of enzyme activity are a powerful way to demonstrate the functioning of a pathway, they are limited to model species that can be genetically altered, or for which specific mutants are available. Computational modeling of metabolite data and enzyme activities possibly provides a more generally applicable, alternative way to elucidate Pro regulation in response to various conditions.

Predicting the effect of future climate conditions on Pro metabolism is complicated by the multitude of environmental variables involved. For instance, Pro accumulated in *Arapidopsis* plants in response to drought, but it did not respond to combined drought and heat stress (Rizhsky *et al*., 2004). Similarly, drought and UV radiation increased Pro content in durum wheat, but not in the presence of elevated CO_2_ (Balouchi *et al*., 2009). Because the effects of combined perturbations of different environmental factors are not always additive, it is necessary to investigate their interaction and combined impact to understand the effect of future climate scenarios (Rizhsky *et al*., 2004; Miller *et al*., 2009). The extent of Pro accumulation also varies among plant species (Maggio *et al*., 2002; Reddy *et al*., 2004; Kishor *et al*., 2005). For instance, *Thellungiella halophila* accumulates two‐ to three‐fold more Pro than observed in *Arabidopsis thaliana* under control and salt stress (Kant *et al*., 2006). It is therefore important to study changes in Pro synthesis in various species under future climate conditions, including agronomically and ecologically relevant crops.

In order to gain a deeper mechanistic understanding of the regulation of Pro metabolism under drought stress in future climate scenarios, we performed measurements at multiple organisational levels, and combined the experimental data with a pathway‐control computational modelling approach. We compare responses and regulatory strategies in species from two agronomically important plant families – grasses and legumes.

## Materials and Methods

### Experimental setup and plant harvest

A mesocosm experiment was conducted at the Drie Eiken Campus of Antwerp University, Belgium. Temperate grassland species – two grasses (*Lolium perenne* L., *Poa pratensis* L.) and two N‐fixing legumes (*Medicago lupulina* L., *Lotus corniculatus* L.) – were chosen, based on their general occurrence, CO_2_ sensitivity (C_3_ species), small size and common soil requirements (all seeds from Herbiseed, Twyford, UK). Plants were grown for 4 months in 16 sunlit, temperature and CO_2_‐controlled chambers. Each chamber contained two populations (nine individuals) of each species, grown in PVC tubes (19 cm diameter, 40 cm height) with sandy soil (96% sand, pH 7.6). The soil initially contained 1.3% carbon, 19 mg nitrate‐nitrogen (N), 1.1 mg ammonium‐N, 13 mg phosphorus (P) k^−1^ air dry soil (De Boeck *et al*., 2011), and was not additionally fertilized. Nodules were present (but not quantified) on the legume roots, indicating active N fixation. Previous work using identical substrates and species, showed that soil N content was hardly affected over the time of the experiment (Van den Berge *et al*., 2011).

As the primary aim of the study was to investigate the effect of CO_2_ on the water deficit stress and elevated temperature, and not the CO_2_ effect by itself, and because of space limitations, we opted for an incremental design of climate conditions maintaining sufficient (four) replicates. Climate conditions and treatments were: A, current climate, with ambient air temperature (*T*_air_) and CO_2_ and sufficient water; D, drought stress in ambient climate; DT, drought stress in a warming climate (*T*_air_ + 3°C); and DTC, drought stress in a future climate (*T*_air_ + 3°C, and elevated CO_2_ at 615 ± 81 ppm). The climate scenarios were chosen according to the IPCC‐SRES B2‐scenario prediction of moderate change for the year 2100 (Murray & Ebi, 2012). For more details about microclimate parameters/growth conditions, see AbdElgawad *et al*. (2014, 2015).

Drought stress was induced by withdrawal of irrigation, at 122 days after sowing, when plants of all species were already flowering. Aboveground biomass (FW, 4 cm above soil surface) was harvested at the onset of visual stress symptoms (leaf discoloration, wilting and dehydration) in 50% of the population. At the same time, monitoring the reduction in photosynthesis rates and stomatal conductance demonstrated that plants experienced considerable and similar stress levels (AbdElgawad *et al*., 2014). Harvest time was therefore 1 wk in *M. lupulina* and *L. corniculatus*, 2 wk in *L. perenne* and 3 wk in *P. pratensis*. Plant material was frozen into liquid nitrogen, immediately after harvest, and stored at −80°C until analysis.

### Amino acid measurements

Amino acids were extracted by homogenizing plant shoots (200 mg FW) in 1 ml of 80% (v/v) aqueous ethanol (MagNALyser; Roche, Vilvoorde, Belgium; 1 min, 7000 rpm), spiked with norvaline to estimate the loss of amino acids during extraction, and centrifugation at 20 000 ***g*** for 20 min. The supernatant was vacuum‐evaporated, and the pellet resuspended in 1 ml of chloroform. The plant residue was re‐extracted with 1 ml HPLC grade water using MagNALyser and the supernatant after centrifugation (20 000 ***g*** for 20 min) was mixed with the pellet suspended in chloroform. Then the extracts were centrifuged for 10 min at 20 000 ***g*** and the aqueous phase was filtered by Millipore microfilters (0.2‐μm pore size) before assaying amino acid concentrations. Amino acids were measured by using a Waters Acquity UPLC‐tqd system (Milford, MA, USA) equipped with a BEH amide 2.1 × 50 column (Sinha *et al*., 2013).

### Enzyme activity assays

Enzyme activities were measured according to described procedures, without modifications. All measurements were scaled down for semi‐high throughput analysis using a microplate reader (Synergy Mx; Biotek Instruments Inc., Winooski, VT, USA). Assays were optimized to obtain linear time and protein‐concentration dependence. In short, the methods were as follows. For GS (EC: 6.3.1.2), P5CS, P5CR (EC: 1.5.1.2) and ProDH (EC: 1.5.99.8), tissue was extracted (100 mg ml^−1^ 50 mM Tris‐HCl, pH 7.4, 2% (w/v) polyvinylpyrrolidone, 4 mM DTT, 10 mM MgC12, 1 mM EDTA, 10% glycerol and 2 mM PMSF) (Zhang *et al*., 1995; Temple *et al*., 1996; Lutts *et al*., 1999) in a MagNALyser (Roche, 3 × 20 s). GS activity was determined in Tris‐acetate reaction buffer (Tris‐acetate, 200 mM, pH 6.4), monitoring the accumulation of γ‐glutamyl hydroxamate (*A*_500_, Temple *et al*., 1996). P5CS activity (assayed in 50 mM tris‐HCl pH 7.0) was monitored as accumulation of γ‐glutamyl hydroxamate (*A*_535_, Zhang *et al*., 1995). P5CR was determined in the same reaction buffer by measuring the P5C‐dependent oxidation of NADH (*A*_340_, Lutts *et al*., 1999). ProDH was assayed in Tris‐HCl buffer (200 mM, pH 8.0, Sakuraba *et al*., 2001), by measuring the reduction of 2,6‐dichloroindophenol (DCIP) (*A*_600_) caused by proline oxidation. For ARG (EC: 3.5.3.1) and OAT (EC: 2.6.1.13) measurements, tissue was extracted in a 50 mM potassium phosphate buffer pH 7.0, containing 2% (w:v) polyvinylpyrrolidone, 1 mM EDTA, 15% glycerol, 2 mM PMSF and 10 mM 2‐mercaptoethanol (100 mg ml^−1^, MagNALyser). ARG activity was measured in glycine‐NaOH, (100 mM, pH 10.0) by measuring the urea produced (Nuzum & Snodgrass, 1976). OAT activity was measured in the same Tris‐HCl reaction mixture as ProDH, measuring the reduction of NADH (A340, Charest & Ton Phan, 1990). Enzyme activities were calculated using molar absorption coefficients. For each reaction 250–500 μg of protein was used. Protein concentrations in the plant extracts were determined by Lowry *et al*. (1951), using BSA as a standard.

### RNA extraction and quantitative (Q)‐PCR

For total RNA extraction, *L. perenne* and *M. lupulina* shoot material was homogenized (MagNaLyser) and RNA was extracted from the homogenates using the RNeasy Plant Mini Kit (Qiagen). The RNA quantity and integrity was measured using a high resolution gel cartridge on a *QIAxcel* platform (Qiagen). A starting amount of 1 μg RNA was transcribed to first‐strand cDNA (Maxima^®^ First Strand cDNA Synthesis Kit; Fermentas, Hinxton, UK). mRNA expression in *L. perenne* and *M. lupulina* shoots exposed to D, DT and DTC was compared with that in ambient conditions by Q‐PCR using the primers listed in Supporting Information Table S1. For the design of the primers, we used very highly conserved regions of the genes encoding these enzymes in multiple species (details in Methods S1). QPCR analyses were performed on an Mx3000P QPCR System (Agilent, Cedar Creek, TX, USA). A SuperMix‐UDG (Invitrogen) was used to perform QPCR analysis (denaturation: 10 min at 95°C; amplification and quantification: 40 times, 40 s at 95°C, 20 s at 55°C, 30 s at 72°C). Melt curve analyses of the target genes and reference genes were performed, which resulted in single products with specific melting temperatures. In addition, ‘no‐template’ controls (i.e. with water) were run to ensure no contamination of reagents and no primer–dimer formation. Glyceraldehyde 3‐phosphate dehydrogenase (GAPDH) and β‐actin 2 for *M. lupulina* and elongation factor‐4α (elf‐4α) and ubiquitin C (UBC) for *L. perenne*; GAPDH and elf‐4α were chosen for *M. lupulina* and *L. perenne*, respectively, as the most stable genes across the samples and these were used as endogenous standards to calculate relative mRNA expression by the standard curve method. Standard curves were generated by serial dilution of a random mixture of control samples.

### Monte Carlo‐driven metabolic control analysis

Because detailed reaction kinetics of the Pro biosynthesis pathways are not available, we developed a method that makes maximal use of the available information to understand pathway control. The method employs knowledge of the pathway topology, the (measured) metabolite concentrations, the pathway stoichiometry and a generic form of enzyme kinetics to calculate control coefficients (Table 1; see later Fig.  5; see later Methods S3). Such control coefficients are defined in metabolic control theory/analysis as the relative change in an output variable of a pathway operating at steady‐state (e.g. metabolite concentration or flux), in response to a (small) relative change in a reaction rate (enzyme activity). Control coefficients represent perfect to relatively accurate predictions of pathway behaviour for infinitesimal and finite perturbations, respectively (Fell, 1997).

**Table 1:** Overview of the stoichiometric numbers of the pathway model reactions

	ARG	OAT	GDH	GOGAT	GS	P5CS	P5CR	PRODH	PROCO	AKGPR	GLUPR	P5CDH
Orn	1	−1	0	0	0	0	0	0	0	0	0	0
akG	0	−1	1	−1	0	0	0	0	0	1	0	0
Glu	0	1	−1	2	−1	−1	0	0	0	0	1	1
Gln	0	0	0	−1	1	0	0	0	0	0	0	0
P5c	0	1	0	0	0	1	1	1	0	0	0	−1
Pro	0	0	0	0	0	0	−1	−1	−1	0	0	0

The rows specify the stoichiometric numbers of the pathway model variables (metabolites: Orn, Ornithine; akG, α‐ketoglutarate; Glu, glutamate; Gln, glutamine; P5c, D1‐pyrroline‐5‐carboxylate; Pro, proline) relative to the various reactions in the columns (ARG, arginase; OAT, ornithine d‐aminotransferase; GDH, glutamate dehydrogenase; GOGAT, glutamate synthase; GS, glutamine synthetase; P5CS, pyrroline‐5‐carboxylate synthetase; P5CR, pyrroline‐5‐carboxylate reductase; PRODH, proline dehydrogenase; PROCO, proline consumption; AKGPR, α‐ketoglutarate production; GLUPR, glutamate production; P5CDH, pyrroline‐5‐carboxylate dehydrogenase; cf. Supporting Information Table S2).

The Pro concentration [Pro] control coefficient is defined as:

$$C_{v_{i}}^{\left[\mathrm{Pro}\right]}=\frac{\partial \ln[\text{Pro}]}{\partial \ln v_{i}}=\frac{v_{i}\partial \left[\text{Pro}\right]}{\left[\text{Pro}\right]\partial v_{i}}≅\frac{\left({\left[\text{Pro}\right]}_{p}-\left[\text{Pro}\right]\right)}{\left[\text{Pro}\right]}/\frac{\left(E_{i,p}-E_{i}\right)}{E_{i}}$$

(*v*_*i*_, reaction rate; *E*_*i*_, enzyme concentration of reaction *i* in the reference steady‐state). The subscript *p* refers to the new steady‐state after perturbing the enzyme activity (directly proportional to concentration) as indicated earlier. Control coefficients can be derived by inversion of the Elasticity or E matrix, which depends on the pathway's stoichiometric relations and elasticities.

Elasticities are derivatives describing the dependence of the enzyme rates on their substrates and products. These are ‘local’ (instead of pathway‐level) properties that depend on the enzyme's individual kinetic equations (including the kinetic constants). Our strategy to estimate elasticities was to use a generalized type of rate law (Liebermeister *et al*., 2010), for (rapid equilibrium) reversible enzyme mechanisms (cf. Methods S3). From this, the following expressions were derived for the corresponding elasticities:

$$\begin{array}{cc} & \varepsilon_{S_{i}}^{v}=n_{i}\left[\frac{1}{1-\rho }-\frac{\alpha_{i}{\left(1+\alpha_{i}\right)}^{n_{i}-1}\prod_{j\neq i}{\left(1+\alpha_{j}\right)}^{n_{j}}}{D}\right]\\ & \varepsilon_{P_{i}}^{v}=-n_{i}\left[\frac{\rho }{1-\rho }+\frac{\pi_{i}{\left(1+\pi_{i}\right)}^{n_{i}-1}\prod_{j\neq i}{\left(1+\pi_{j}\right)}^{n_{j}}}{D}\right] \end{array}$$

(α_*i*_, concentration of enzyme substrate *i* relative to the *K*_*m*_ value for that metabolite; π_*j*_, concentration of enzyme product *j* relative to the *K*_*m*_ value for that product; *n*_*i*_, stoichiometric coefficient of species *i*; $\rho =\frac{\Gamma^{\prime }}{K_{eq}^{\prime }}$, mass‐action ratio with $\Gamma^{\prime }=\frac{\prod_{j}\pi_{j}}{\prod_{i}\alpha_{i}}$; Γ′, mass‐action ratio under biochemical reference conditions); correspondingly $K_{\mathrm{eq}}^{\prime }$ the equilibrium constant; *D*, denominator of the Liebermeister rate law without allosteric regulation (Methods S3)).

For instance for the sensitivity of GOGAT with respect to Glu this would mean:

$$\varepsilon_{\mathrm{Glu}}^{\mathrm{GOGAT}}=-2\left[\frac{\rho_{\mathrm{GOGAT}}}{1-\rho_{\mathrm{GOGAT}}}+\frac{\pi_{\mathrm{Glu}}\left(1+\pi_{\mathrm{Glu}}\right)\left(1+\pi_{\mathrm{NADP}}\right)}{\left(1+\alpha_{\mathrm{akG}}\right)\left(1+\alpha_{\mathrm{Gln}}\right)\left(1+\alpha_{\mathrm{NADPH}}\right)+{\left(1+\pi_{\mathrm{Glu}}\right)}^{2}\left(1+\pi_{\mathrm{NADP}}\right)-1}\right]$$

(Methods S2 contains all required expressions).

In order to determine the required elasticities, the Γ values were calculated with the measured or estimated metabolite and cofactor levels (details in Table S3) and the *K*_eq_ value of the corresponding reactions calculated based on tabulated or estimated values of free energies of formation (Methods S3). For the unknown α and π values and the *J*_*i*_ ratios of the elasticity matrix, we performed a Monte Carlo sampling, randomly selecting values over realistic intervals (Methods S3). Each resulting *E* matrix was then inverted to derive the so‐called control matrix *C*, containing all relevant control coefficients (Reder, 1988). The ensemble of control matrices resulting from the Monte Carlo sampling was then used to plot the Pro concentration control distribution and calculate the reported median and 10 and 90 percentiles over 1000 Monte Carlo runs (a sufficient number for convergence of the statistics).

### Statistical analysis

Data were tested for homogeneity of variance and normality; transformations were not necessary. Results were analysed by one‐way ANOVA, using SPSS 16.0 statistical software (SPSS Inc., Chicago, IL, USA), and significant differences between the means were determined by using the Tukey test (*P *<* *0.05) (*n *=* *4).

## Results

### Pro synthesis through the Glu pathway

**Figure 2:**
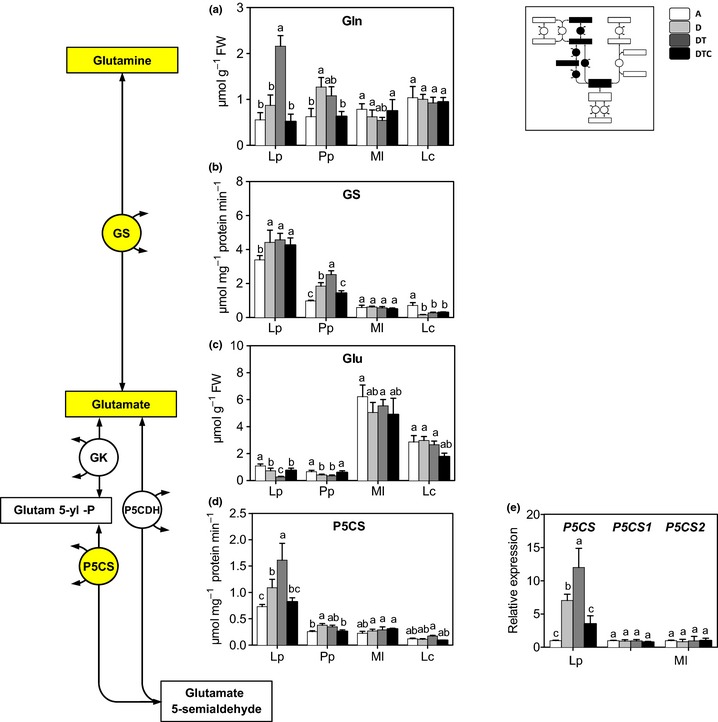
Glutamate (Glu) pathway: changes in transcript levels, enzyme activity and metabolic concentrations in four grassland species, *Lolium perenne* (Lp), *Poa pratensis* (Pp), *Medicago lupulina* (Ml) and *Lotus corniculatus* (Lc). Plants were grown at: A, ambient CO_2_ and temperature (*T*) and sufficient water; D, ambient CO_2_ and *T* and drought stress; DT, ambient CO_2_ with elevated *T* and drought stress; and DTC, elevated CO_2_ and *T* and drought stress. Panels show concentrations of (a) glutamine (Gln) and (c) Glu, (b) activity of glutamine synthetase (GS), and (d, e) activity and expression level of P5C synthase (P5CS). Different letters in the graph represent significant differences between the four treatments (error bars, ± SE. Tukey test; *P *<* *0.05; *n *=* *4). The inset shows the relative position of the results in the overall pathway. GK, glutamate 5‐kinase; P5CDH, pyrroline‐5‐carboxylate dehydrogenase.

Drought stress alone (D) or in combination with elevated temperature (DT), significantly affected Gln (increase) and Glu (decrease) content in the grasses (*P. pratensis* and *L. perenne*), but not in the legumes (*M. lupulina* and *L. corniculatus*; Fig. 2a,c). Increasing CO_2_ (DTC) reduced the stress impact in the grass species, but had no effect in the legumes. Consistent with the changes in Gln and Glu concentrations, GS activity increased under stress treatment in *L. perenne* and *P. pratensis*, but not in the legumes (Fig. 2b).

Glu is converted to Glu 5‐semialdehyde by GK and P5CS (Fig. 1), which spontaneously converts to P5C. The activity of P5CS was induced by drought and elevated temperature, in the grass species but not in the legumes (Fig. 2d). In parallel, stress conditions increased the transcriptional level of the *P5CS* gene in *L. perenne*, but not in *M. lupulina* (Fig. 2e) (transcription levels were not determined for *P. pratensis* and *L. corniculatus*). Elevated CO_2_ significantly reduced the stress effect in P5CS, at both the enzyme activity and the transcriptional levels.

### Pro synthesis through the Orn pathway

**Figure 3:**
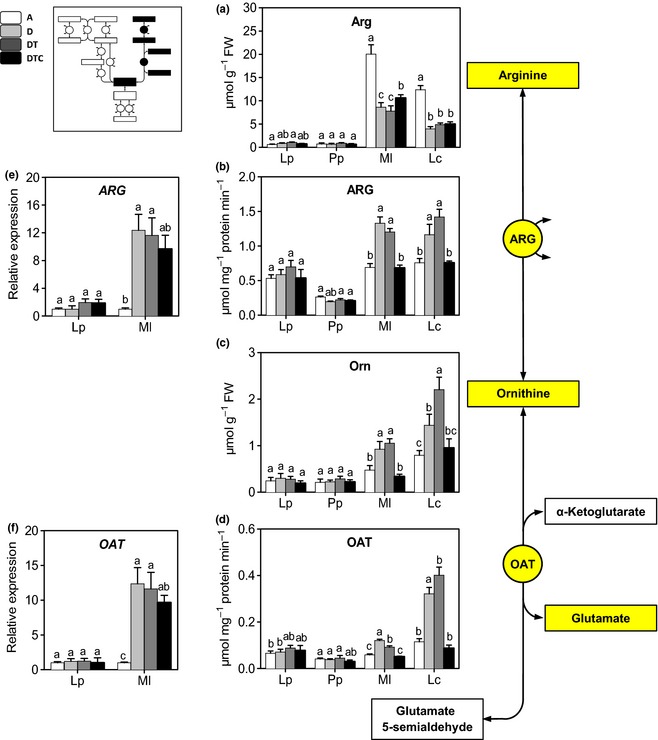
Ornithine pathway: changes in transcript levels, enzyme activity and metabolic concentrations in the four‐grassland species, *Lolium perenne* (Lp), *Poa pratensis* (Pp), *Medicago lupulina* (Ml) and *Lotus corniculatus* (Lc). Plants were grown at: A, ambient CO_2_ and temperature (*T*) and sufficient water; D, ambient CO_2_ and *T* and drought stress; DT, ambient CO_2_, elevated *T* and drought stress; and DTC, elevated CO_2_ and *T* and drought stress. Panels show concentrations of (a) Arg, (c) ornithine (Orn), (b, e) activity and expression level of arginase (ARG), and (d, f) ornithine‐d‐aminotransferase (OAT). Different letters in the graph represent significant differences between the four treatments (error bars, ± SE. Tukey test; *P *<* *0.05; *n *=* *4). The inset shows the relative position of the results in the overall pathway.

Arg provides a second precursor that feeds into the Pro synthesis pathway via Orn. In contrast to Glu and Gln, the concentrations of Arg and Orn were considerably higher in the legumes compared with the grasses. Drought and warming had no significant effects on Arg or Orn concentrations in the grass species (Fig. 3a,c), whereas Arg significantly decreased and Orn concentrations significantly increased in the legumes. The lower concentrations of Arg and increased concentrations of Orn are consistent with increased ARG activity under stress conditions (Fig. 3b). Also *ARG* transcript levels (in *M. lupulina*) increased under stress (Fig. 3e). Elevated CO_2_ reduced the stress effect on ARG activity and expression in *M. lupulina* and *L. corniculatus*. OAT activity and expression levels also increased under stress conditions in the legumes, but not in the grass species, and this effect was reversed by elevated CO_2_ (Fig. 3f).

### The P5C–Pro metabolism cycle

**Figure 4:**
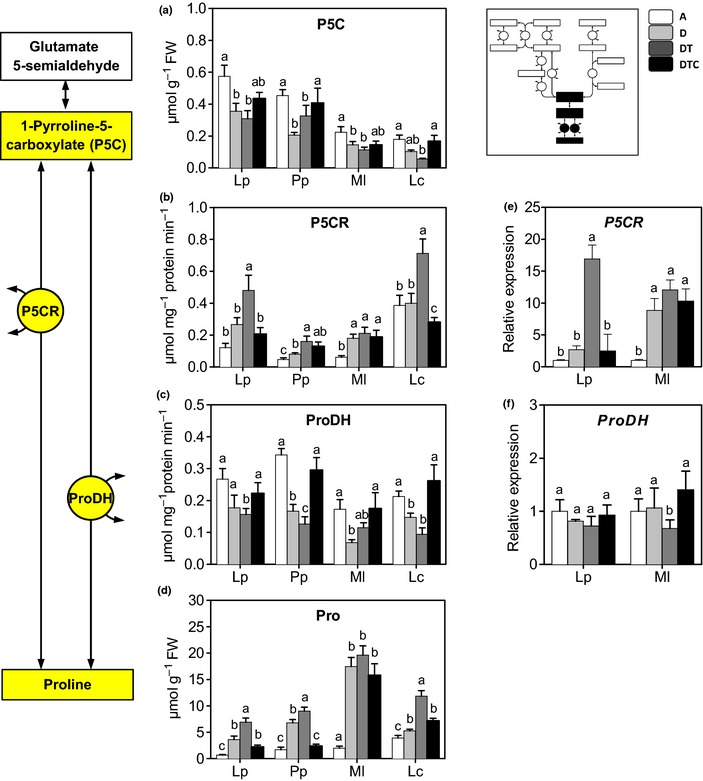
Δ1‐pyrroline‐5‐carboxylate (P5C)–proline (Pro) cycle. Changes in transcript levels, enzymes activity and metabolic concentrations in four‐grassland species, *Lolium perenne* (Lp), *Poa pratensis* (Pp), *Medicago lupulina* (Ml) and *Lotus corniculatus* (Lc). Plants were grown at: A, ambient CO_2_ and temperature (*T*) and sufficient water; D, ambient CO_2_ and *T* and drought stress; DT, ambient CO_2_, elevated *T* and drought stress; and DTC, elevated CO_2_ and *T* and drought stress. Panels show concentrations of (a) P5C and (d) Pro, (b, e) activity and expression level of P5C reductase (P5CR), and (c, f) proline dehydrogenase (ProDH). Different letters in the graphs represent significant differences between the four treatments (error bars, ± SE. Tukey test; *P *<* *0.05; *n *=* *4). The inset shows the relative position of the results in the overall pathway.

The Glu pathway and the Orn pathway converge via GSA on the P5C–Pro cycle. In the absence of stress, Pro concentrations were similar in all species (0.1–0.3 μmol g^−1^ FW, Fig. 4d). Drought and elevated temperature generally caused pronounced increases in Pro content and concomitant decreases in its immediate precursor P5C. In most cases elevated CO_2_ reduced the stress‐induced Pro increase. Final Pro concentrations from P5C (Fig. 4a) are the net result of the activity of P5CR, P5CDH (not measured), ProDH, and Pro consumption. Changes in the Pro profiles correlated relatively well with changes in the P5CR activity in most species (Fig. 4b). Changes in P5CR activity also matched closely with changes in *P5CR* transcripts (data only for *L. perenne* and *M. lupulina*, Fig. 4e). Stress‐induced P5CR activity was reversed in elevated CO_2_. ProDH activity, controlling Pro oxidation to P5C, showed opposite effects to P5CR in response to drought, elevated temperature and CO_2_ (Fig. 4c). Its activity decreased under stress, and this effect was prevented under elevated CO_2_. *ProDH* transcripts varied largely in parallel with the enzyme activity (Fig. 4f).

In summary, stress treatments strongly induced Pro accumulation and elevated CO_2_ counters this effect. In the grass species, increased Pro concentrations originate mainly through the Glu pathway, whereas in legumes the Orn pathway is responsible. Elevated CO_2_ decreased the stress impact essentially at the activity level of all enzymes involved.

### Thermodynamics and metabolic control analysis

**Figure 5:**
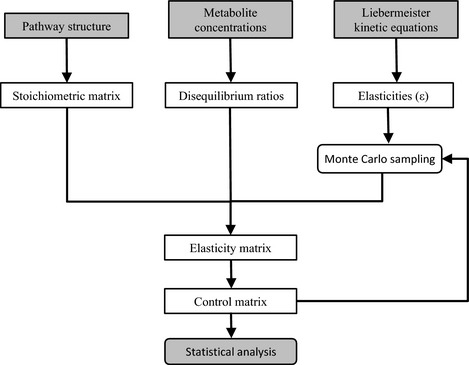
Schematic representation of the Monte Carlo metabolic control analysis. The grey rectangles represent required input information, which is translated in a different form in the first steps of the procedure to yield an elasticity matrix. Matrix inversion is then used to produce a control matrix. The elasticity matrix is iteratively generated via a Monte Carlo sampling of saturation ratios and pathway flux ratios (for details see the Materials and Methods section).

Based on a newly developed computational method we derived estimates of control coefficients, which quantify the effect of regulating the activity of each of the enzymes in the pathway on the Pro concentration. This method uses pathway structure, thermodynamic information and generic reaction kinetics, combined with Monte Carlo sampling (Fig. 5; for a detailed description cf. Methods S3).

A crucial determinant of the control calculations is the distance from equilibrium of the reactions as expressed by their so‐called disequilibrium ratio. Apart from the measured (and in some cases estimated, cf. Table S3) metabolite concentrations (or concentration ratios), this requires the reactions' equilibrium constants (*K*_eq_ values). For that purpose molar reaction free energies were calculated ($\Delta_{r}G^{\prime }$; Tables 2, S4; cf. Methods S3) which specify the direction of the net (positive) flux. Their negative values indicated that nearly all reactions are spontaneous in the expected direction. Two exceptions are the ARG and ProDH reactions for which the disquilibrium ratio was adjusted (see Methods S3).

**Table 2:** Thermodynamic properties and estimates of control coefficients of pathway reactions

Reaction^a^	Enzyme^b^	$\Delta G_{r}^{0\prime }$ c (kJ mol^−1^)	$\Delta G_{r}^{\prime }$ d (kJ mol^−1^)		(Pro)‐control coefficient Median (*P*_10_; *P*_90_)^e^	
			Lp	Ml	Lp	Ml
	ARG	66.2	43	36	0.051 (0.023; 0.11)	0.05 (0.023; 0.12)
	OAT	−26.9	−20	−20	0.1 (0.054; 0.26)	0.11 (0.056; 0.24)
	GS	−22.8	−28	−32	−0.056 (−0.28; −0.009)	−0.053 (−0.29; 0.0098)
	GOGAT	−51.4	−44	−36	−0.017 (−0.098; −0.0016)	−0.014 (−0.086; −0.0013)
	GDH^f^	−38.1	−11	−7	−0.085 (−0.19; −0.032)	−0.079 (−0.18; −0.033)
	P5CS	−8.33	−29	−36	0.056 (−0.0035; 0.25)	0.039 (−0.013; 0.24)
	P5CDH	−27.8	−32	−25	−0.016 (−0.098; 0.00083)	−0.012 (−0.086; 0.0039)
	P5CR	−31.1	−29	−24	0.069 (0.011; 0.25)	0.064 (0.0059; 0.25)
	PRODH	31	25	20	−0.021 (−0.097; −0.0032)	−0.02 (−0.086; −0.0016)
α‐k‐Gla‐supply					0.77 (0.020; 2.4)	0.71 (0.095; 2.3)
Glu‐supply					0.31 (−0.18; 1.4)	0.25 (−0.20; 1.3)
Pro‐consumption					−0.96 (−0.99; −0.87)	−0.97 (−1.0; −0.88)

a cf. Figs 1, 6.

b For enzyme names cf. Fig. 1 and the Materials and Methods section.

c $\Delta G_{r}^{0\prime }$ the reaction free energy under biochemical standard conditions.

d $\Delta G_{r}^{\prime }$ the reaction free energy adjusted for the experimental metabolite concentrations.

e Median, 10^th^ and 90^th^ percentile values corresponding to the Pro concentration control coefficient distributions from *L. perenne* (Fig. S1) and *M. Lupulina* (Fig. S2).

f The glutamate dehydrogenase (GDH) reaction is taken positive in the direction of glutamate (Glu) production.

α‐k‐Gla, α‐ketoglutarate; ARG, arginase; OAT, ornithine d‐aminotransferase; GS, glutamine synthetase; GOGAT, glutamate synthase; P5CS, pyrroline‐5‐carboxylate synthetase; P5CDH, pyrroline‐5‐carboxylate dehydrogenase; P5CR, pyrroline‐5‐carboxylate reductase; PRODH, proline dehydrogenase.

**Figure 6:**
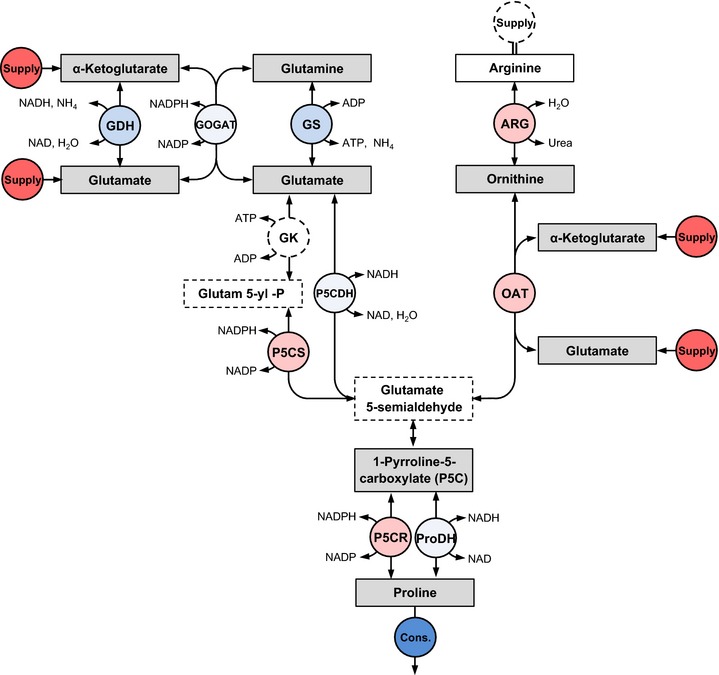
Modeled proline (Pro) pathway. Model variables are depicted in grey rectangular boxes, whereas the reactions (Table 2) are in circles colored according to the calculated control coefficients with red and blue colors indicating (respectively) positive and negative control (color intensity based on the highest of the absolute values of 10^th^ or 90^th^ percentiles from Table 2: brightest red/blue if maximum value > 0.3, weaker if maximum value > 0.1, otherwize a nearly white color). Dashed lines indicate biochemical entities not explicit in the model. The Arg influx is represented as a constant source with varying arginase (ARG) activities accounting for the variation in input. GDH, NADH‐glutamate dehydrogenase; GOGAT, glutamate synthase; GS, glutamine synthetase; GK, glutamate 5‐kinase; P5C, Δ1‐pyrroline‐5‐carboxylate; P5CS, P5C synthase; P5CDH, pyrroline‐5‐carboxylate dehydrogenase; P5CR, P5C reductase; PRODH, proline dehydrogenase; ARG, arginase; OAT, ornithine‐d‐aminotransferase.

**Figure 7:**
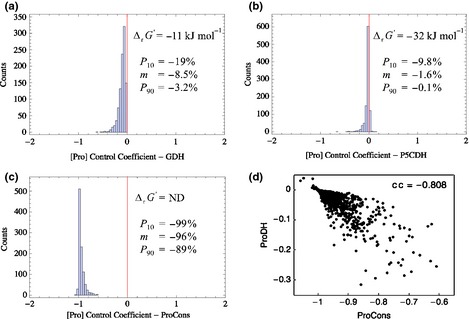
Predicted control properties of (*Lolium* sp.) proline (Pro) pathway enzymes. (a) Histogram describing the control coefficient distribution for NADH‐glutamate dehydrogenase (GDH). The negative reaction free energy ($\Delta_{r}G^{\prime }$) implies a net positive reaction flux in the sense of Pro production; however, the negative range of the control distribution (characterized by its 10th‐percentile (*P*_10_), median (*m*) and ninetieth percentile (*P*_90_) values) indicates that GDH upregulation will reduce the Pro concentration. (b) Histogram describing the control coefficient distribution for pyrroline‐5‐carboxylate dehydrogenase (P5CDH). The negative reaction free energy ($\Delta_{r}G^{\prime }$) implies a net negative reaction flux in the sense of Pro breakdown, the control coefficient values indicates a small negative influence on the Pro concentration. (c) Histogram describing the control coefficient distribution for Pro consumption (ProCons) indicating a strong negative influence on the Pro concentration. (d) Plot of proline dehydrogenase (ProDH) vs Pro consumption control coefficient values for all individual Monte Carlo simulation runs. The corresponding correlation coefficient (cc) shows a strong negative correlation with less negative values for Pro consumption control typically associated with a more pronounced negative control by ProDH.

Our calculations yielded a set of (Pro) concentration control coefficient distributions (one per reaction step, cf. Figs S1, S2 for *L. perenne* and *M. lupulina*) reflecting how modulating the respective pathway enzymes potentially affects the Pro concentration (Fig. 6). The distributions are approximately mono‐modal and we have represented them by their median values, P_10_ and P_90_ percentiles (Tables 2, S3). The Orn pathway has in general a strong potential for Pro upregulation (P_90_ values up to 12 and 26%, respectively). Conversely, increasing GS activity is predicted to negatively affect Pro (P_10_ as low as −29%). Although it is thermodynamically driven to produce Glu, GDH is predicted to negatively impact Pro concentrations (P_10_ as low as −19%, Table 2; Fig. 7a), which likely originates from its competition with OAT for α‐ketoglutarate (α‐*k*‐Gla). GOGAT and P5CDH appear less suitable for regulatory purposes with −9.8% as the most pronounced P_10_ value, and median values close to zero (Table 2; Fig. 7b). The downstream‐located P5CS and P5CR again are more likely to be Pro control sites with P_90_ values of *c*. 25%. The level of (negative) control exerted by ProDH appears to be limited (P_10_ values not higher than −9.7%). Control calculations for a larger distance from equilibrium, for instance due to high NAD : NADH and Pro : P5CR ratios in the mitochondrial compartment yield roughly similar values. Importantly, our model suggests a negative effect of increasing (additional) Pro consumption (with median values near −100%, Fig. 7c) and a (broadly) positive effect of increasing α‐*k*‐Gla (median values up to 77%) and Glu (median values up to 31%) supplies on Pro concentration.

In order to better understand how control by different reactions is related, we calculated correlation coefficients for all pairs of control coefficient (cc) distributions (Table S5). Sequential reactions in the principal branches such as the Orn pathway (e.g. cc of 0.64 and 0.63 for *L. perenne* and *M. lupulina* in the current climate, respectively), and P5CS‐P5CR (cc of 0.60 and 0.49 in *L. perenne* and *M. lupulina*, respectively), are correlated, indicating that they share the control of that branch. Similarly, GS tends to have a more negative impact on Pro concentrations if P5CS has a more positive impact (cc of −0.66 and −0.64 for *L. perenne* and *M. lupulina*). Enzymes catalysing opposite reactions such as P5CS‐P5CDH (cc of −0.79 and −0.76 for *L. perenne* and *M. lupulina*), and P5CR‐ProDH (cc of −0.69 and −0.76 for *L. perenne* and *M. lupulina*) tend to have positively correlated absolute values of their Pro control coefficients. Interestingly, control on the Pro consumption step negatively correlated with the ProDH (cc of −0.81 and −0.80 for *L. perenne* and *M. lupulina*; Table S5; Fig. 7d) and P5CR (cc of −0.63 and −0.52 for *L. perenne* and *M. lupulina*) steps. On the one hand, a more negative influence of Pro consumption is associated with a less negative influence of ProDH and P5CDH. On the other, for P5CR a more negative influence of Pro consumption is associated with a less positive influence of this step (cc of 0.68 and 0.71 for *L. perenne* and *M. lupulina*).

Taken together, our control calculations indicate the potential for regulatory changes of ARG, OAT, GS, P5CS and P5CR to significantly influence Pro concentration. The correlations indicate that coregulation through several of these steps is possible and would be more effective than regulation of a single step.

## Discussion

In order to understand the regulation of Pro metabolism, under climate change scenarios, contrasting grassland plant species were subjected to drought and temperature stress and elevated atmospheric CO_2_, and Pro metabolism was analysed at the metabolic, enzyme activity and transcriptional levels. Because it is impossible to intuitively connect regulatory changes in metabolites and activities to the effective change of Pro concentrations, we developed a computational approach, which directly quantifies this causal relationship as control coefficients. The lack of kinetic information on the enzymes was circumvented in part by a Monte Carlo sampling over realistic parameter ranges, using a generic type of reversible enzyme kinetics. Furthermore, we made use of the available information about the metabolite concentrations by translating them into thermodynamic properties, which could be fed into kinetic expressions. This method allowed the calculation of control coefficients for all enzymes and precursors to the pathway, expressing their potential impact on Pro concentrations.

### Drought and elevated temperature induce concerted changes in Pro metabolism

Individual effects of drought and high temperature on Pro have been studied and it has been shown that Pro accumulates under these stresses (Parida *et al*., 2008; Szabados & Savouré, 2010; Krasensky & Jonak, 2012). However, relatively little is known about how their combination alters Pro content. Drought significantly increased Pro content in all species we investigated, and additional temperature elevation generally increased the stress impact, as has been previously observed (De Ronde *et al*., 2004; Simon‐Sarkadi *et al*., 2005), The temperature‐induced changes indicate that even a relatively small increase in growth temperature, applied during the full life cycle, can significantly affect the response of Pro metabolism to drought.

Elevated CO_2_ reduced the stress impact on Pro concentrations, as has been also observed previously (Balouchi *et al*., 2009; Pérez‐López *et al*., 2010). We found that this CO_2_ effect is the result of repressing Pro biosynthesis, as well as activating its degradation. Also our computational estimation of pathway control points to a concerted regulation of anabolic and catabolic pathways. Moreover, these effects occur not only at the metabolite and enzyme activity levels, but also at the transcript level. The fact that elevated CO_2_ causes a general reversal of multiple drought‐induced regulatory changes, suggests that it impacts a common signal controlling the drought response across the whole pathway or upstream from changes in Pro metabolism.

An explanation for such an effect may lie in reduced H_2_O_2_ formation, as a result of suppression of photorespiration by elevated CO_2_ (Ainsworth & Rogers, 2007; Jia *et al*., 2010; Salazar‐Parra *et al*., 2012; Zinta *et al*., 2014; AbdElgawad *et al*., 2015). H_2_O_2_, induced by stress treatments, may act as a signal that activates stress‐response pathways (Gill & Tuteja, 2010). Verslues *et al*. (2007) reported that increased H_2_O_2_ may induce Pro biosynthesis by affecting abscisic acid (ABA) concentrations. H_2_O_2_ treatment also activated Pro biosynthesis by increasing the activity and transcriptional levels of P5CS, ARG and OAT and decreasing the activity of ProDH in maize seedlings (Geissler *et al*., 2009; Yang *et al*., 2009). Reduced H_2_O_2_ formation could therefore be involved in the stress and CO_2_ effects on Pro synthesis.

Elevated CO_2_ also increases Pro catabolism (ProDH activity) Pro catabolism may be an important energy source (Kishor *et al*., 2005; Szabados & Savouré, 2010; Liang *et al*., 2013). Pro degradation provides reducing potential to the mitochondria for production of up to 30 ATP/Pro (Atkinson, 1977; Kishor *et al*., 2014). This process provides high‐energy output to resume growth after stress (Verbruggen *et al*., 1996; Hare & Cress, 1997).

### The role of the Glu or Orn pathways in Pro accumulation, differs between grasses and legumes, and is possibly affected by N status

Despite the limitation that we have only studied four species, from two clades, and at a single developmental time point, our results quite clearly indicate that in the legumes the Orn pathway (ARG and OAT) is activated under drought stress, whereas in the grasses drought activates the Glu pathway (P5CS). From the literature it is also clear that the contribution of each pathway in the stress‐induced Pro responses varies with species (Pardha Saradhi & Mohanty, 1997; Reddy *et al*., 2004; Kishor *et al*., 2005) and stress exposure (Xue *et al*., 2009; Yang *et al*., 2009). For example, no significant role in Pro accumulation was observed for the Orn pathway in *Brassica napus* and *Vigna aconitifolia*, whereas it was activated together with the Glu pathway in *A. thaliana* (Roosens *et al*., 1998) and *M. truncatula* (Armengaud *et al*., 2004).

The predominant involvement of the Orn pathway may also be related to plant N status. Increased N input induced OAT expression, possibly via accumulation of Orn or Arg (Delauney *et al*., 1993). Increased OAT activity was also implicated in facilitating N recycling from Arg to Glu (Funck *et al*., 2008). In addition, leaf N content was not affected by the drought and CO_2_ treatments in the grass species, but increased in the legumes (AbdElgawad *et al*., 2014). Therefore, the predominant activation of the Orn pathway in legumes under drought stress, may be closely related to their N‐fixing ability. It would be of interest to further explore the relation between N content and Pro concentrations, by varying N fertilization in grasses and legumes.

### Control analysis indicates potential mechanisms for Pro regulation

Finding causal relations between changes in enzyme activities and changes in metabolite concentrations (such as Pro) is not straightforward, even for a linear pathway. Indeed, it has been demonstrated that enzymes of a pathway have different levels of control on the global pathway, determined by the location of the corresponding reaction in the pathway, how far it is from equilibrium and its kinetics. Control coefficients are a useful aid in quantifying the level of control (Kacser & Burns, 1979; Uys *et al*., 2007; Rohwer, 2012; Tang *et al*., 2012). To determine concentration control coefficients for all reaction steps, a complete and detailed kinetic model of the pathway is required (Fell, 1997). For Pro biosynthesis the structure of the pathway is well known (Fig. 1), but the kinetic equations of the respective enzymes are relatively poorly characterized (Verslues & Sharma, 2010). We have circumvented this limitation by applying metabolic control analysis (Kacser & Burns, 1979; reviewed in Morgan & Rhodes, 2002) in a new way using generic kinetics. This allows the prediction of which enzyme activity changes are most effective, and therefore more likely responsible, for the increase in Pro concentrations under drought and elevated temperature. However, the structural and thermodynamic constraints imposed by the network topology and reaction thermodynamics are not strong enough to explain the more subtle differences in the control between species and conditions of this study.

In general our analysis of correlations between control coefficients demonstrates that concerted changes of enzyme activities are more effective than single changes and are probably necessary considering that we observed up to 10‐fold changes in Pro concentrations between conditions. This provides an explanation for the multitude of observed regulatory changes under stress conditions. Control coefficients calculated by Monte Carlo sampling of generic kinetic constants predict that for all studied species, the increased P5CR, and to a lesser extent decreased ProDH, were responsible for increased Pro concentrations. Furthermore, for the grasses the increased P5CS and for the legumes increased ARG and OAT activities, contributed to that effect. Because our findings are robust to variations in metabolite concentrations, modifying the activities of ARG, OAT, P5CS and P5CR can be predicted to be the most effective strategy to manipulate cellular Pro concentrations (Hmida‐Sayari *et al*., 2005; Miller *et al*., 2009; You *et al*., 2012). Experimentally, this is supported by the observed regulation of ARG, OAT and P5CR for legumes, and P5CS and P5CR for grasses under different perturbations.

A powerful way to confirm the validity of a model lies in manipulating, genetically or pharmacologically, the activity of particular enzymes and analyse the metabolic effect. Notably, for Pro biosynthesis a significant number of experiments have been performed on plants with reduced or increased expression of Pro metabolism enzymes. For example P5CS has been altered in *A. thaliana*, but also in *Glycine max*,* Medicago trucatula*,* Nicotiana tabacum* and others. Also for ProDH, GS, OAT and P5CDH, lines with altered expression are available. A compilation of these studies (Table S6) demonstrates that the large majority of the experimental outcomes match the change predicted on the basis of our control analysis. These results therefore provide considerable independent experimental validation for the computational analysis. Regarding the discrepancies it should be noted that not all experimental results are mutually consistent (for instance for P5CS). In principle, a multitude of explanations can be postulated to explain aberrant control properties, for instance based on allosteric regulation (leading to feed‐back or feed‐forward relations; see, for instance, Fell & Snell, 1988; Hofmeyr & Olivier, 2002) not modelled by our generic approach or compensatory expression and activity changes in response to the genetic perturbations. Basic additions or alterations to the model pathway such as adding an extra glutamine branch or modifying the kinetics of the branches could not resolve those issues.

An important aspect of the *in vivo* Pro pathway is, indeed, its connection to other metabolic pathways. Within the assumptions of our model a strong positive influence of αkGla and Glu supply and a strong negative influence of Pro consumption were identified. Kinetic parameter regimes with a less pronounced negative influence from Pro consumption tend to be characterized by significantly more control on some of the reactions upstream from proline like P5CR, ProDH and P5CDH. This inverse relationship could be instrumental in restoring Pro concentrations if the consumption rate is increased (for example increased protein synthesis under conditions of fast growth). To limit the number of unknown parameters, we have taken a minimalistic approach and selected the model variables in accordance with the available data. Extensions and refinements to the model are possible, such as integrating reactions of the urea cycle and other pathways of amino acid metabolism. Provided that the enzyme topology and metabolite concentrations are known, the method can in principle also be applied to other metabolic pathways.

### Conclusion

It is clear that future climate conditions, such as drought and elevated temperature, are likely to negatively impact the growth of grasslands, and the associated feed and food production. By way of protection, Pro concentrations are strongly induced in all tested species, although through different mechanisms, and possibly directed by the plant N status. Ecological differences in the capacity of plants to assimilate N, may therefore affect Pro metabolism in adverse environmental conditions. Notably, elevated CO_2_ nearly abolished the stress responses, by affecting both Pro biosynthesis and degradation. Computational analysis indicates that pathway regulation is obtained by simultaneous changes in multiple enzymes.

## Supporting information

Please note: Wiley Blackwell are not responsible for the content or functionality of any supporting information supplied by the authors. Any queries (other than missing material) should be directed to the *New Phytologist* Central Office.

**Fig. S1** Proline concentration control distribution of the *Lolium perenne* Pro pathway metabolite data under current climate conditions.
**Fig. S2** Proline concentration control distribution of the *Medicago lupulina* Pro pathway metabolite data under current climate conditions.
**Table S1** Real‐time PCR targets, GenBank identifier and primers for transcript analysis of references genes and genes involved in proline metabolisms
**Table S2 **Overview of enzymes included in the computational model, with name, abbreviation, Enzyme Commission number and biochemical reaction scheme
**Table S3 **Free energies of formation and cellular metabolite concentrations used in the calculation of disequilibrium ratios
**Table S4 **Extended version of Table 2 with additional calculated values for *Poa pratensis* and *Lotus corniculatus* under ambient conditions
**Table S5** Correlation analysis with correlation coefficients tabulated for the *Lolium* and *Medicago* control distributions (Figs S1 and S2, respectively)
**Table S6** Comparison of changes in proline concentrations in plants with altered proline‐biosynthesis enzymes, to changes expected on the basis of the proposed model
**Methods S1** Primers design for Q‐PCR.
**Methods S2 **Liebermeister kinetics based elasticity expressions used for metabolic control analysis.
**Methods S3 **Free energy calculations and Monte Carlo‐based metabolic control analysis.

## References

[nph13481-bib-0001] AbdElgawad H , Farfan‐Vignolo ER , de Vos D , Asard H . 2015 Elevated CO_2_ mitigates drought and temperature‐induced oxidative stress differently in grasses and legumes. Plant Science 231: 1–10. 25575986 10.1016/j.plantsci.2014.11.001

[nph13481-bib-0002] AbdElgawad H , Peshev D , Zinta G , Van den Ende W , Janssens IA , Asard H . 2014 Climate extreme effects on the chemical composition of temperate grassland species under ambient and elevated CO_2_: a comparison of fructan and non‐fructan accumulators. PLoS ONE 9: e92044. 24670435 10.1371/journal.pone.0092044 PMC3966776

[nph13481-bib-0500] Abraham E , Rigo G , Szekely G , Nagy R , Konez C , Szabados L . 2003 Light dependent induction of proline biosynthesis by abscisic acid and salt stress is inhibited by brassinosteriods in Arabidopsis. Plant Molecular Biology 51: 363–372. 12602867 10.1023/a:1022043000516

[nph13481-bib-0003] Ainsworth EA , Rogers A . 2007 The response of photosynthesis and stomatal conductance to rising [CO_2_]: mechanisms and environmental interactions. Plant, Cell & Environment 30: 258–270. 10.1111/j.1365-3040.2007.01641.x 17263773

[nph13481-bib-0004] Armengaud P , Thiery L , Buhot N , Grenier‐De March G , Savoure A . 2004 Transcriptional regulation of proline biosynthesis in *Medicago truncatula* reveals developmental and environmental specific features. Physiologia Plantarum 120: 442–450. 15032841 10.1111/j.0031-9317.2004.00251.x

[nph13481-bib-0005] Atkinson DE . 1977 Cellular energy metabolism and its regulation. New York, NY, USA: Academic Press.

[nph13481-bib-0006] Balouchi H , Sanavy SM , Emam Y , Dolatabadian A . 2009 UV radiation, elevated CO_2_ and water stress effect on growth and photosynthetic characteristics in durum wheat. Plant, Soil & Environment 55: 443–453.

[nph13481-bib-0007] Charest C , Ton Phan C . 1990 Cold acclimation of wheat (*Triticum aestivum*): properties of enzymes involved in proline metabolism. Physiologia Plantarum 80: 159–168.

[nph13481-bib-0501] De Boeck HJ , Dreesen FE , Janssens IA , Nijs I . 2011 Whole‐system responses of experimental plant communities to climate extremes imposed in different seasons. New Phytologist 189: 806–817. 21054412 10.1111/j.1469-8137.2010.03515.x

[nph13481-bib-0008] De Ronde J , Cress W , Krüger G , Strasser R , Van Staden J . 2004 Photosynthetic response of transgenic soybean plants, containing an *Arabidopsis P5CR* gene, during heat and drought stress. Journal of Plant Physiology 161: 1211–1224. 15602813 10.1016/j.jplph.2004.01.014

[nph13481-bib-0009] Delauney AJ , Hu CA , Kishor PB , Verma DP . 1993 Cloning of ornithine delta‐aminotransferase cDNA from *Vigna aconitifolia* by trans‐complementation in *Escherichia coli* and regulation of proline biosynthesis. Journal of Biological Chemistry 268:18673–18678. 8103048

[nph13481-bib-0010] Fell D . 1997 Understanding the control of metabolism. London, UK: Portland Press.

[nph13481-bib-0011] Fell DA , Snell K . 1988 Control analysis of mammalian serine biosynthesis. Feedback inhibition on the final step. Biochemical Journal 256: 97–101. 2851987 10.1042/bj2560097 PMC1135373

[nph13481-bib-0012] Feng GQ , Li Y , Cheng ZM . 2014 Plant molecular and genomic responses to stresses in projected future CO_2_ environment. Critical Reviews in Plant Sciences 33: 238–249.

[nph13481-bib-0013] Funck D , Stadelhofer B , Koch W . 2008 Ornithine‐delta‐aminotransferase is essential for arginine catabolism but not for proline biosynthesis. BMC Plant Biology 8: 40. 18419821 10.1186/1471-2229-8-40 PMC2377265

[nph13481-bib-0014] Geissler N , Hussin S , Koyro H‐W . 2009 Interactive effects of NaCl salinity and elevated atmospheric CO_2_ concentration on growth, photosynthesis, water relations and chemical composition of the potential cash crop halophyte *Aster tripolium* L. Environmental and Experimental Botany 65: 220–231.

[nph13481-bib-0015] Gill SS , Tuteja N . 2010 Reactive oxygen species and antioxidant machinery in abiotic stress tolerance in crop plants. Plant Physiology and Biochemistry 48: 909–930. 20870416 10.1016/j.plaphy.2010.08.016

[nph13481-bib-0016] Hare P , Cress W . 1997 Metabolic implications of stress‐induced proline accumulation in plants. Plant Growth Regulation 21: 79–102.

[nph13481-bib-0502] Hmida‐Sayari A , Gargouri‐Bouzid R , Bidani A , Jaoua L , Savouré A , Jaoua S . 2005 Overexpression of ▵1‐pyrroline‐5‐carboxylate synthetase increases proline production and confers salt tolerance in transgenic potato plants. Plant Science 169: 746‐752.

[nph13481-bib-0017] Hofmeyr JH , Olivier BG . 2002 The regulatory design of an allosteric feedback loop: the effect of saturation by pathway substrate. Biochemical Society Transactions 30: 19–25. 12023817 10.1042/0300-5127:0300019

[nph13481-bib-0018] Hong Z , Lakkineni K , Zhang Z , Verma DPS . 2000 Removal of feedback inhibition of Δ1‐pyrroline‐5‐carboxylate synthetase results in increased proline accumulation and protection of plants from osmotic stress. Plant Physiology 122: 1129–1136. 10759508 10.1104/pp.122.4.1129 PMC58947

[nph13481-bib-0019] Hu C , Delauney AJ , Verma D . 1992 A bifunctional enzyme (delta 1‐pyrroline‐5‐carboxylate synthetase) catalyzes the first two steps in proline biosynthesis in plants. Proceedings of the National Academy of Sciences, USA 89: 9354–9358. 10.1073/pnas.89.19.9354 PMC50125 1384052

[nph13481-bib-0020] Huang Z , Zhao L , Chen D , Liang M , Liu Z , Shao H , Long X . 2013 Salt stress encourages proline accumulation by regulating proline biosynthesis and degradation in Jerusalem artichoke plantlets. PLoS ONE 8: e62085. 23637970 10.1371/journal.pone.0062085 PMC3639250

[nph13481-bib-0021] IPCC . 2012 Field CB, Barros V, Stocker TF, Qin D, Dokken DJ, Ebi KL, Mastrandrea MD, Mach KJ, Plattner G‐K, Allen SK *et al*, eds. Managing the risks of extreme events and disasters to advance climate change adaptation: a special report of Working Groups I and II of the Intergovernmental Panel on Climate Change. Summary for policymakers. Cambridge, UK: Cambridge University Press, 1–19.

[nph13481-bib-0022] Jia Y , Tang S , Wang R , Ju X , Ding Y , Tu S , Smith DL . 2010 Effects of elevated CO_2_ on growth, photosynthesis, elemental composition, antioxidant level, and phytochelatin concentration in *Lolium mutiforum* and *Lolium perenne* under Cd stress. Journal of Hazardous Materials 180: 384–394. 20439132 10.1016/j.jhazmat.2010.04.043

[nph13481-bib-0023] Kacser H , Burns J . 1979 Molecular democracy: who shares the controls? Biochemal Society Transactions 7: 1149–1160. 10.1042/bst0071149 389705

[nph13481-bib-0024] Kant S , Kant P , Raveh E , Barak S . 2006 Evidence that differential gene expression between the halophyte, *Thellungiella halophila*, and *Arabidopsis thaliana* is responsible for higher levels of the compatible osmolyte proline and tight control of Na^+^ uptake in *T. halophila* . Plant, Cell & Environment 29: 1220–1234. 10.1111/j.1365-3040.2006.01502.x 17080945

[nph13481-bib-0025] Kim GB , Nam YW . 2013 A novel Delta(1)‐pyrroline‐5‐carboxylate synthetase gene of *Medicago truncatula* plays a predominant role in stress‐induced proline accumulation during symbiotic nitrogen fixation. Journal of Plant Physiology 170: 291–302. 23158502 10.1016/j.jplph.2012.10.004

[nph13481-bib-0026] Kishor K , Polavarapu B , Sreenivasulu N . 2014 Is proline accumulation *per se* correlated with stress tolerance or is proline homeostasis a more critical issue? Plant, Cell & Environment 37: 300–311. 10.1111/pce.12157 23790054

[nph13481-bib-0027] Kishor PK , Hong Z , Miao G‐H , Hu C‐AA , Verma DPS . 1995 Overexpression of [delta]‐pyrroline‐5‐carboxylate synthetase increases proline production and confers osmotolerance in transgenic plants. Plant Physiology 108: 1387–1394. 12228549 10.1104/pp.108.4.1387 PMC157516

[nph13481-bib-0028] Kishor PK , Sangam S , Amrutha R , Laxmi PS , Naidu K , Rao K , Rao S , Reddy K , Theriappan P , Sreenivasulu N . 2005 Regulation of proline biosynthesis, degradation, uptake and transport in higher plants: its implications in plant growth and abiotic stress tolerance. Current Science 88: 424–438.

[nph13481-bib-0029] Kiyosue T , Yoshiba Y , Yamaguchi‐Shinozaki K , Shinozaki K . 1996 A nuclear gene encoding mitochondrial proline dehydrogenase, an enzyme involved in proline metabolism, is upregulated by proline but downregulated by dehydration in *Arabidopsis* . Plant Cell 8: 1323–1335. 8776899 10.1105/tpc.8.8.1323 PMC161248

[nph13481-bib-0030] Krasensky J , Jonak C . 2012 Drought, salt, and temperature stress‐induced metabolic rearrangements and regulatory networks. Journal of Experimental Botany 63: 1593–1608. 22291134 10.1093/jxb/err460 PMC4359903

[nph13481-bib-0031] Li X , Ahammed G , Zhang Y , Zhang G , Sun Z , Zhou J , Zhou Y , Xia X , Yu J , Shi K . 2014 Carbon dioxide enrichment alleviates heat stress by improving cellular redox homeostasis through an ABA‐independent process in tomato plants. Plant Biology 17: 81–89. 24985337 10.1111/plb.12211

[nph13481-bib-0032] Liang X , Zhang L , Natarajan SK , Becker DF . 2013 Proline mechanisms of stress survival. Antioxidants & Redox Signaling 19: 998–1011. 23581681 10.1089/ars.2012.5074 PMC3763223

[nph13481-bib-0033] Liebermeister W , Uhlendorf J , Klipp E . 2010 Modular rate laws for enzymatic reactions: thermodynamics, elasticities and implementation. Bioinformatics 26: 1528–1534. 20385728 10.1093/bioinformatics/btq141

[nph13481-bib-0034] Lowry OH , Rosebrough NJ , Farr AL , Randall RJ . 1951 Protein measurement with the Folin phenol reagent. Journal of Biological Chemistry 193: 265–275. 14907713

[nph13481-bib-0035] Lutts S , Majerus V , Kinet JM . 1999 NaCl effects on proline metabolism in rice (*Oryza sativa*) seedlings. Physiologia Plantarum 105: 450–458.

[nph13481-bib-0036] Maggio A , Miyazaki S , Veronese P , Fujita T , Ibeas JI , Damsz B , Narasimhan ML , Hasegawa PM , Joly RJ , Bressan RA . 2002 Does proline accumulation play an active role in stress‐induced growth reduction? Plant Journal 31: 699–712. 12220262 10.1046/j.1365-313x.2002.01389.x

[nph13481-bib-0037] Mani S , Van de Cotte B , Van Montagu M , Verbruggen N . 2002 Altered levels of proline dehydrogenase cause hypersensitivity to proline and its analogs in *Arabidopsis* . Plant Physiology 128: 73–83. 11788754 PMC148945

[nph13481-bib-0038] Miller G , Honig A , Stein H , Suzuki N , Mittler R , Zilberstein A . 2009 Unraveling delta1‐pyrroline‐5‐carboxylate‐proline cycle in plants by uncoupled expression of proline oxidation enzymes. Journal of Biological Chemistry 284: 26 482–26 492. 10.1074/jbc.M109.009340 PMC2785336 19635803

[nph13481-bib-0039] Morgan JA , Rhodes D . 2002 Mathematical modeling of plant metabolic pathways. Metabolic Engineering 4: 80–89. 11800577 10.1006/mben.2001.0211

[nph13481-bib-0503] Murray V , Ebi KL . 2012 IPCC special report on managing the risks of extreme events and disasters to advance climate change adaptation (SREX). Journal of Epidemiology and Community Health 66: 759–760. 22766781 10.1136/jech-2012-201045

[nph13481-bib-0040] Nanjo T , Fujita M , Seki M , Kato T , Tabata S , Shinozaki K . 2003 Toxicity of free proline revealed in an *Arabidopsis* T‐DNA‐tagged mutant deficient in proline dehydrogenase. Plant and Cell Physiology 44: 541–548. 12773641 10.1093/pcp/pcg066

[nph13481-bib-0041] Naudts K , Van den Berge J , Farfan E , Rose P , AbdElgawad H , Ceulemans R , Janssens I , Asard H , Nijs I . 2014 Future climate alleviates stress impact on grassland productivity through altered antioxidant capacity. Environmental and Experimental Botany 99: 150–158.

[nph13481-bib-0042] Nuzum CT , Snodgrass PJ . 1976 Multiple assays of the five urea‐cycle enzymes in human liver homogenates In: Grisolia S, Baguena R, Mayor F, eds. The urea cycle. New York, NY, USA: John Wiley & Sons, 325–349.

[nph13481-bib-0043] Pandey R , Zinta G , AbdElgawad H , Ahmad A , Jain V , Janssens IA . 2015 Physiological and molecular alterations in plants exposed to high [CO_2_] under phosphorus stress. Biotechnology Advances 33: 303–316. 25797341 10.1016/j.biotechadv.2015.03.011

[nph13481-bib-0044] Pardha Saradhi P , Mohanty P . 1997 Involvement of proline in protecting thylakoid membranes against free radical‐induced photodamage. Journal of Photochemistry and Photobiology B: Biology 38: 253–257.

[nph13481-bib-0045] Parida AK , Dagaonkar VS , Phalak MS , Aurangabadkar LP . 2008 Differential responses of the enzymes involved in proline biosynthesis and degradation in drought tolerant and sensitive cotton genotypes during drought stress and recovery. Acta Physiologiae Plantarum 30: 619–627.

[nph13481-bib-0046] Pérez‐López U , Robredo A , Lacuesta M , Muñoz‐Rueda A , Mena‐Petite A . 2010 Atmospheric CO_2_ concentration influences the contributions of osmolyte accumulation and cell wall elasticity to salt tolerance in barley cultivars. Journal of Plant Physiology 167: 15–22. 19660829 10.1016/j.jplph.2009.06.019

[nph13481-bib-0047] Phang J . 1985 The regulatory functions of proline and pyrroline‐5‐carboxylic acid. Current Topics in Cellular Regulation 25: 91–132. 2410198 10.1016/b978-0-12-152825-6.50008-4

[nph13481-bib-0048] Reddy AR , Chaitanya KV , Vivekanandan M . 2004 Drought‐induced responses of photosynthesis and antioxidant metabolism in higher plants. Journal of Plant Physiology 161: 1189–1202. 15602811 10.1016/j.jplph.2004.01.013

[nph13481-bib-0049] Reder C . 1988 Metabolic control theory: a structural approach. Journal of Theoretical Biology 135: 175–201. 3267767 10.1016/s0022-5193(88)80073-0

[nph13481-bib-0050] Rizhsky L , Liang H , Shuman J , Shulaev V , Davletova S , Mittler R . 2004 When defense pathways collide. The response of *Arabidopsis* to a combination of drought and heat stress. Plant Physiology 134: 1683–1696. 15047901 10.1104/pp.103.033431 PMC419842

[nph13481-bib-0051] Rohwer JM . 2012 Kinetic modelling of plant metabolic pathways. Journal of Experimental Botany 63: 2275–2292. 22419742 10.1093/jxb/ers080

[nph13481-bib-0052] Roosens NH , Al Bitar F , Loenders K , Angenon G , Jacobs M . 2002 Overexpression of ornithine‐δ‐aminotransferase increases proline biosynthesis and confers osmotolerance in transgenic plants. Molecular Breeding 9: 73–80.

[nph13481-bib-0053] Roosens NH , Thu TT , Iskandar HM , Jacobs M . 1998 Isolation of the ornithine‐δ‐aminotransferase cDNA and effect of salt stress on its expression in *Arabidopsis thaliana* . Plant Physiology 117: 263–271. 9576796 10.1104/pp.117.1.263 PMC35011

[nph13481-bib-0054] Roosens NH , Willem R , Li Y , Verbruggen I , Biesemans M , Jacobs M . 1999 Proline metabolism in the wild‐type and in a salt‐tolerant mutant of *Nicotiana plumbaginifolia* studied by ^13^C‐nuclear magnetic resonance imaging. Plant Physiology 121: 1281–1290. 10594115 10.1104/pp.121.4.1281 PMC59495

[nph13481-bib-0055] Sakuraba H , Takamatsu Y , Satomura T , Kawakami R , Ohshima T . 2001 Purification, characterization, and application of a novel dye‐linked L‐proline dehydrogenase from a *hyperthermophilic archaeon*,* Thermococcus profundus* . Applied and Environmental Microbiology 67: 1470–1475. 11282592 10.1128/AEM.67.4.1470-1475.2001 PMC92756

[nph13481-bib-0056] Salazar‐Parra C , Aguirreolea J , Sánchez‐Díaz M , Irigoyen JJ , Morales F . 2012 Climate change (elevated CO_2_, elevated temperature and moderate drought) triggers the antioxidant enzymes' response of grapevine cv. Tempranillo, avoiding oxidative damage. Physiologia Plantarum 144: 99–110. 21929631 10.1111/j.1399-3054.2011.01524.x

[nph13481-bib-0057] Savoure A , Jaoua S , Hua XJ , Ardiles W , Van Montagu M , Verbruggen N . 1995 Isolation, characterization, and chromosomal location of a gene encoding the delta 1‐pyrroline‐5‐carboxylate synthetase in *Arabidopsis thaliana* . FEBS Letters 372: 13–19. 7556633 10.1016/0014-5793(95)00935-3

[nph13481-bib-0058] Simon‐Sarkadi L , Kocsy G , Várhegyi Á , Galiba G , de Ronde JA . 2005 Genetic manipulation of proline accumulation influences the concentrations of other amino acids in soybean subjected to simultaneous drought and heat stress. Journal of Agricultural and Food Chemistry 53: 7512–7517. 16159180 10.1021/jf050540l

[nph13481-bib-0059] Sinha AK , Giblen T , AbdElgawad H , De Rop M , Asard H , Blust R , De Boeck G . 2013 Regulation of amino acid metabolism as a defensive strategy in the brain of three freshwater teleosts in response to high environmental ammonia exposure. Aquatic Toxicology 130–131: 86–96. 10.1016/j.aquatox.2013.01.003 23384996

[nph13481-bib-0060] Szabados L , Savouré A . 2010 Proline: a multifunctional amino acid. Trends in Plant Science 15: 89–97. 20036181 10.1016/j.tplants.2009.11.009

[nph13481-bib-0061] Szoke A , Miao GH , Hong Z , Verma DP . 1992 Subcellular location of delta‐pyrroline‐5‐carboxylate reductase in root/nodule and leaf of soybean. Plant Physiology 99: 1642–1649. 16669085 10.1104/pp.99.4.1642 PMC1080675

[nph13481-bib-0062] Tang M , Guschina IA , O'Hara P , Slabas AR , Quant PA , Fawcett T , Harwood JL . 2012 Metabolic control analysis of developing oilseed rape (*Brassica napus* cv Westar) embryos shows that lipid assembly exerts significant control over oil accumulation. New Phytologist 196: 414–426. 22901003 10.1111/j.1469-8137.2012.04262.x

[nph13481-bib-0063] Temple SJ , Kunjibettu S , Roche D , Sengupta‐Gopalan C . 1996 Total glutamine synthetase activity during soybean nodule development is controlled at the level of transcription and holoprotein turnover. Plant physiology 112: 1723–1733. 12226474 10.1104/pp.112.4.1723 PMC158106

[nph13481-bib-0064] Uys L , Botha FC , Hofmeyr J‐HS , Rohwer JM . 2007 Kinetic model of sucrose accumulation in maturing sugarcane culm tissue. Phytochemistry 68: 2375–2392. 17555779 10.1016/j.phytochem.2007.04.023

[nph13481-bib-0065] Van den Berge J , Naudts K , Zavalloni C , Janssens I , Ceulemans R , Nijs I . 2011 Altered response to nitrogen supply of mixed grassland communities in a future climate: a controlled environment microcosm study. Plant and Soil 345: 375–385.

[nph13481-bib-0066] Verbruggen N , Hermans C . 2008 Proline accumulation in plants: a review. Amino Acids 35: 753–759. 18379856 10.1007/s00726-008-0061-6

[nph13481-bib-0504] Verslues PE , Sharma S . 2010 Proline metabolism and its implications for plant‐environment interaction. The Arabidopsis Book/American Society of Plant Biologists 8: e0140. 22303265 10.1199/tab.0140 PMC3244962

[nph13481-bib-0067] Verbruggen N , Hua XJ , May M , Van Montagu M . 1996 Environmental and developmental signals modulate proline homeostasis: evidence for a negative transcriptional regulator. Proceedings of the National Academy of Sciences, USA 93: 8787–8791. 10.1073/pnas.93.16.8787 PMC38752 8710950

[nph13481-bib-0068] Verbruggen N , Villarroel R , Van Montagu M . 1993 Osmoregulation of a pyrroline‐5‐carboxylate reductase gene in *Arabidopsis thaliana* . Plant Physiology 103: 771–781. 8022935 10.1104/pp.103.3.771 PMC159047

[nph13481-bib-0069] Verslues PE , Kim YS , Zhu JK . 2007 Altered ABA, proline and hydrogen peroxide in an *Arabidopsis* glutamate:glyoxylate aminotransferase mutant. Plant Molecular Biology 64: 205–217. 17318317 10.1007/s11103-007-9145-z

[nph13481-bib-0070] Wu L , Fan Z , Guo L , Li Y , Chen Z‐L , Qu L‐J . 2005 Over‐expression of the bacterial *nhaA* gene in rice enhances salt and drought tolerance. Plant Science 168: 297–302.

[nph13481-bib-0071] Xue X , Liu A , Hua X . 2009 Proline accumulation and transcriptional regulation of proline biothesynthesis and degradation in *Brassica napus* . BMB reports 42: 28–34. 19192390 10.5483/bmbrep.2009.42.1.028

[nph13481-bib-0072] Yamada M , Morishita H , Urano K , Shiozaki N , Yamaguchi‐Shinozaki K , Shinozaki K , Yoshiba Y . 2005 Effects of free proline accumulation in petunias under drought stress. Journal of Experimental Botany 56: 1975–1981. 15928013 10.1093/jxb/eri195

[nph13481-bib-0073] Yang SL , Lan SS , Gong M . 2009 Hydrogen peroxide‐induced proline and metabolic pathway of its accumulation in maize seedlings. Journal of Plant Physiology 166: 1694–1699. 19446917 10.1016/j.jplph.2009.04.006

[nph13481-bib-0074] Yoshiba Y , Kiyosue T , Nakashima K , Yamaguchi‐Shinozaki K , Shinozaki K . 1997 Regulation of levels of proline as an osmolyte in plants under water stress. Plant, Cell & Physiology 38: 1095–1102. 10.1093/oxfordjournals.pcp.a029093 9399433

[nph13481-bib-0075] You J , Hu H , Xiong L . 2012 An ornithine delta‐aminotransferase gene *OsOAT* confers drought and oxidative stress tolerance in rice. Plant Science 197: 59–69. 23116672 10.1016/j.plantsci.2012.09.002

[nph13481-bib-0076] Zhang C‐S , Lu Q , Verma DPS . 1995 Removal of feedback inhibition of‐pyrroline‐5‐carboxylate synthetase, a bifunctional enzyme catalyzing the first two steps of proline biosynthesis in plants. Journal of Biological Chemistry 270: 20491–20496. 7657626 10.1074/jbc.270.35.20491

[nph13481-bib-0077] Zinta G , AbdElgawad H , Domagalska MA , Vergauwen L , Knapen D , Nijs I , Janssens IA , Beemster GT , Asard H . 2014 Physiological, biochemical, and genome‐wide transcriptional analysis reveals that elevated CO_2_ mitigates the impact of combined heat wave and drought stress in *Arabidopsis thaliana* at multiple organizational levels. Global Change Biology 20: 3670–3685. 24802996 10.1111/gcb.12626

