# Supplementary material for: Grassland species differentially regulate proline concentrations under future climate conditions: an integrated biochemical and modelling approach
Source: New Phytol. 2015 Jun 2;208(2):354–69. doi: 10.1111/nph.13481 (PMC4744684; doi:10.1111/nph.13481)
Supplement: Supplementary file 1 — Fig. S1 Proline concentration control distribution of the Lolium perenne Pro pathway metabolite data under current climate conditions. Fig. S2 Proline concentration control distribution of the Medicago lupulina Pro pathway metabolite data under current climate conditions. Table S1 Real‐time PCR targets, GenBank identifier and primers for transcript analysis of references genes and genes involved in proline metabolisms Table S2 Overview of enzymes included in the computational model, with name, abbreviation, Enzyme Commission number and biochemical reaction scheme Table S3 Free energies of formation and cellular metabolite concentrations used in the calculation of disequilibrium ratios Table S4 Extended version of Table 2 with additional calculated values for Poa pratensis and Lotus corniculatus under ambient conditions Table S5 Correlation analysis with correlation coefficients tabulated for the Lolium and Medicago control distributions (Figs S1 and S2, respectively) Table S6 Comparison of changes in proline concentrations in plants with altered proline‐biosynthesis enzymes, to changes expected on the basis of the proposed model Methods S1 Primers design for Q‐PCR. Methods S2 Liebermeister kinetics based elasticity expressions used for metabolic control analysis. Methods S3 Free energy calculations and Monte Carlo‐based metabolic control analysis. [file NPH-208-354-s001.pdf]

Supporting Information Figs S1 & S2, Tables S1–S6 and Methods S1–S3

Article title: Grassland species differentially regulate proline levels under future climate conditions: an integrated biochemical and modelling approach

Authors: Hamada AbdElgawad, Dirk De Vos, Gaurav Zinta, Malgorzata A. Domagalska, Gerrit T. S. Beemster and Han Asard

Article acceptance date: 13 April 2015

The following Supporting Information is available for this article:

Fig. S1 Proline concentration control distribution of the *Lolium perenne* Pro pathway metabolite data under current climate conditions.

Fig. S2 Proline concentration control distribution of the *Medicago lupulina* Pro pathway metabolite data under current climate conditions.

Table S1 Real-time PCR targets, GenBank identifier, and primers for transcript analysis of references genes and genes involved in proline metabolisms

Table S2 Overview of enzymes included in the computational model, with name, abbreviation, Enzyme Commission number and biochemical reaction scheme

Table S3 Free energies of formation and cellular metabolite concentrations used in the calculation of disequilibrium ratios

Table S4 Extended version of Table 2 with additional calculated values for *Poa pratensis* and *Lotus corniculatus* under ambient conditions

Table S5 Correlation analysis with correlation coefficients tabulated for the *Lolium* and *Medicago* control distributions (Figs S1 and S2, respectively)

Table S6 Comparison of changes in proline levels in plants with altered proline-biosynthesis enzymes, to changes expected on the basis of the proposed model

Methods S1 Primers design for Q-PCR.

Methods S2 Liebermeister kinetics based elasticity expressions used for metabolic control analysis.

Methods S3 Free energy calculations and Monte Carlo-based metabolic control analysis.

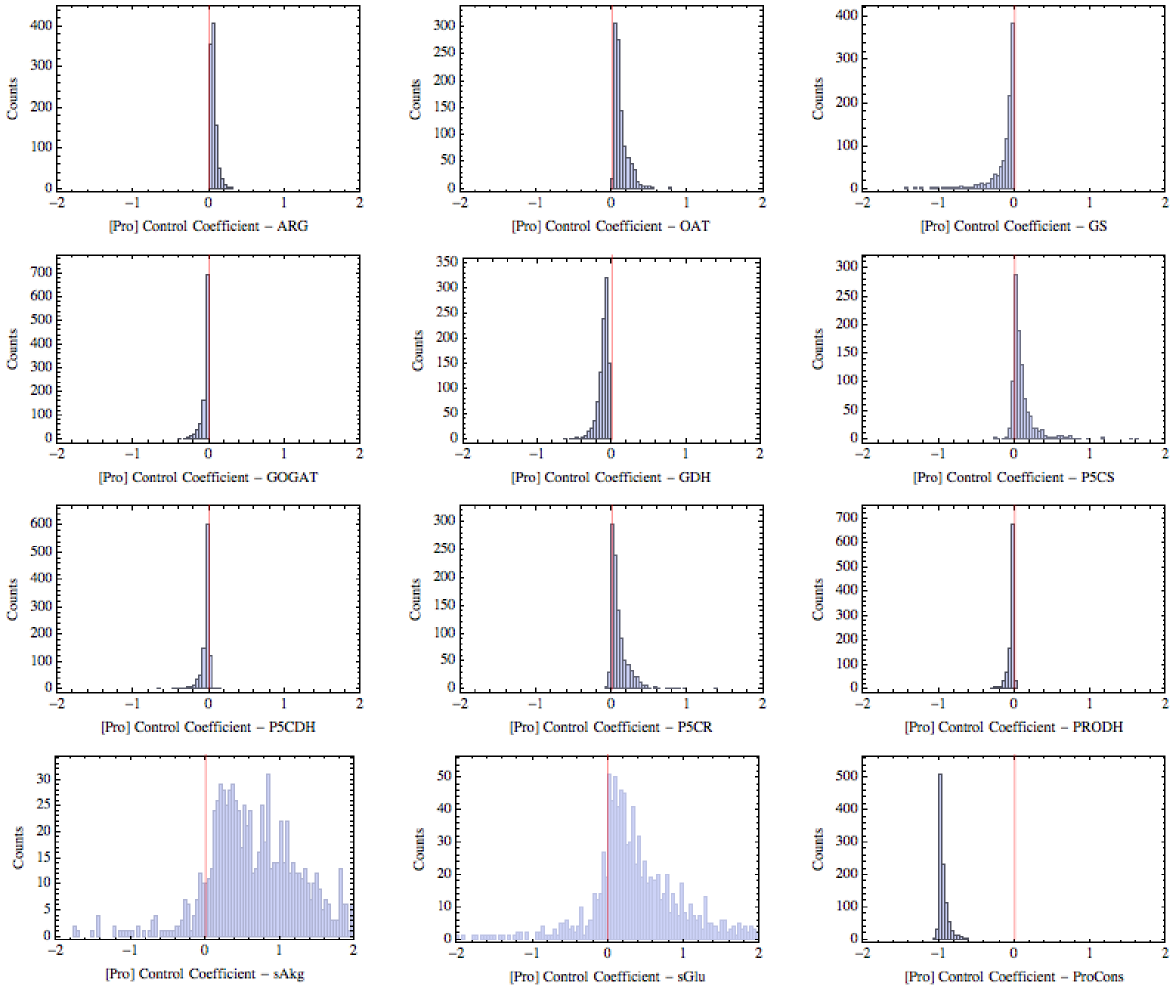

Fig. S1 Proline (Pro) concentration control distribution obtained through Monte Carlo MCA of the *Lolium perenne* Pro pathway metabolite data under current climate conditions.

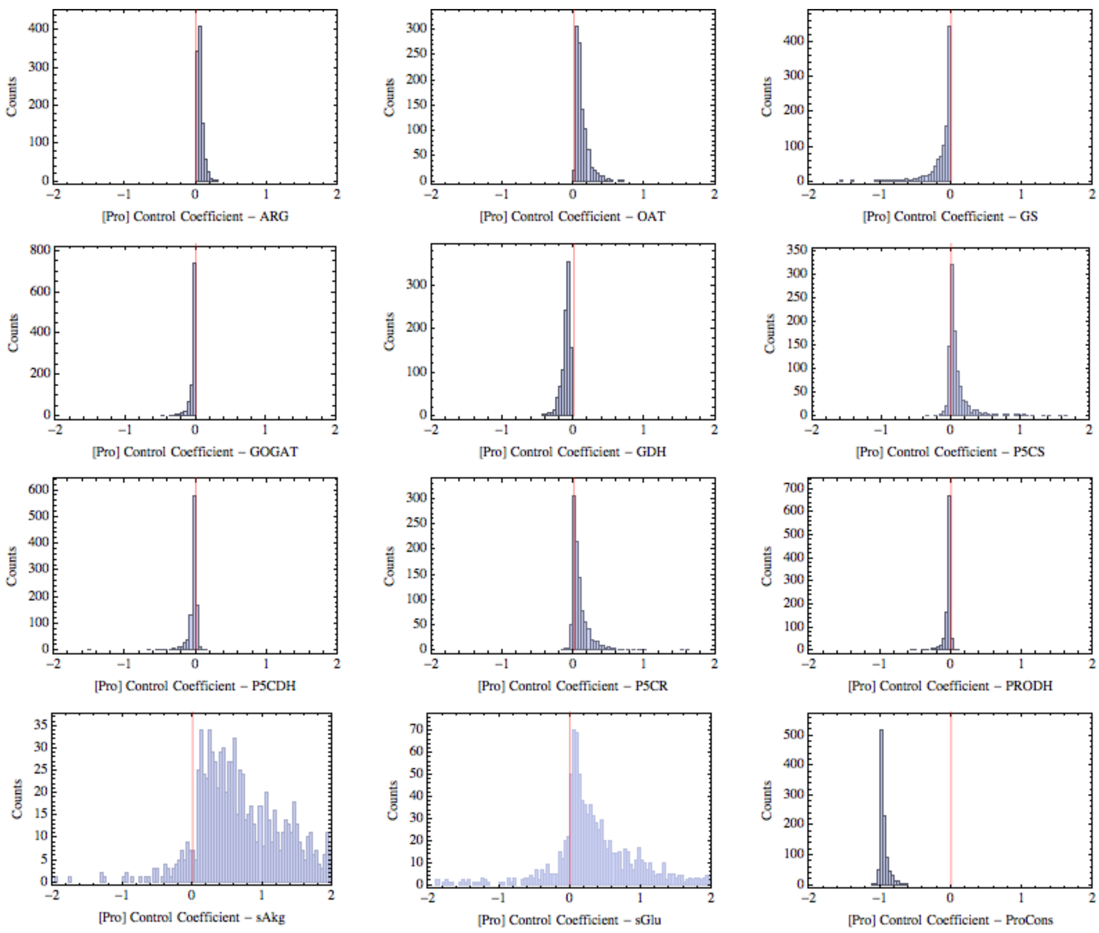

Fig. S2 Proline (Pro) concentration control distribution obtained through Monte Carlo MCA of the *Medicago lupulina* Pro pathway metabolite data under current climate conditions.

Table S1 Real-time PCR targets, GenBank identifier, and primers for transcript analysis of references genes and genes involved in proline metabolisms

**Target description**	**Gen Bank ID**	**Primer sequence (5′→3′)**	**Efficiency (%)**
Lolium-P5CS1	GR515090.1	F: GAGAACAGCGACTCAACC<br>R:GTGACCAGGATCACCTCAT	119.8%
Medicago- P5CS1	AJ278818	F:TCAACGAGAACGACGCA<br>R: GGAGGGCCATTATAAAGACC	83.6%
Medicago- P5CS2	AET87351.1	F: GAGGGCCTTTATAATGGTCC<br>R:TCCTTGAAGCACTCGCA	100.3%
Lolium-Arginase	DT669392.1	F: AGGGCATAACTCATCGTTC<br>R:ATCTTCAACACCGCAGTC	112%
Medicago- Arginase	AES87364.1	F: TTGGAGTTCCTTTGGGTCA<br>R:GGACATCACCAACATCAGT	133.1%
Lolium-OAT	JF747455.1	F: GCTTTGATAGAGCAAGCAGA<br>R:AATAGCTGTTTCCACTCCT	92.2%
Medicago-OAT	CAC82185.1	F: AGCTCACGAGCTTTCTAC<br>R:TGAAGCAGCCACAACAT	103.9%
Lolium-P5CR	BAC15792.1	F: AGCAGCATCAGTGATGT<br>R:AAATATAAGCCGGGCCACTA	106.7%
Medicago- P5CR	AES81244.1	F: GATAGTAACGTCGTCGTCT<br>R:AGCTGTGTTAGGCATTACC	146.1%
Lolium-ProDH	GR520484.1	F: TGCTGGAGTTCGAGGACA<br>R:CGCGCAGAAGTGCTTGTA	101%
Medicago-ProDH	AES77835.1	F: AGGGATTGGAAAGGTGAAC<br>R:CAGCCAACACTCCTCTATT	144.3%
Lolium-eIF-4a	AK073620	F: GGTCGTGTGTTTGACATGCT<br>R:CCTTGAAACCACGAGAAAGC	110%
Medicago- GAPDH	CT573421_3.4	F: TGCCTACCGTCGATGTTTCAGT<br>R:TTGCCCTCTGATTCCTCCTTG	109.4%

Target descriptions for *Lolium* and *Medicago* species genes are based on peptide homologues in corresponding species.

Table S2 Overview of enzymes included in the computational model, with name, abbreviation, Enzyme Commission (EC) number and biochemical reaction scheme

Enzyme name	Abbreviation	EC	Reaction
**Arginase**	**ARG**	3.5.3.1	L-Arg + H_2O $\rightleftarrows$ L-Orn + urea
**Ornithine-aminotranferase**	**OAT**	2.6.1.13	L-Orn + α-k-Gla $\rightleftarrows$ P5C + L-Glu + H_2O
**Glutamine synthetase**	**GS**	6.3.1.2	L-Glu + NH_4 + ATP $\rightleftarrows$ L-Gln + ADP + P_i
**Glutamate synthase**	**GOGAT**	1.4.1.14	L-Gln + α-k-Gla + NADPH $\rightleftarrows$ 2 L-Glu + NADP
**Glutamate dehydrogenase**	**GDH**	1.4.1.2	α -k-Gla + NH_4 + NADH $\rightleftarrows$ L-Glu + NAD + H_2O
**D1-pyrroline-5-carboxylate synthetase**	**P5CS**	#	L-Glu + ATP + NADPH $\rightleftarrows$ P5C + NADP + ADP + P_i + H_2O
**D1-pyrroline-5-carboxylate dehydrogenase**	**P5CDH**	1.5.1.12	P5C + NAD + $2H_2O$ $\rightleftarrows$ L-Glu + NADH
**D1-pyrroline-5-carboxylate reductase**	**P5CR**	1.5.1.2	P5C + NADPH $\rightleftarrows$ L-Pro + NADP
**Proline dehydrogenase**	**PRODH**	1.5.99.8	L-Pro + NAD $\rightleftarrows$ P5C + NADH

We have assumed G5-SA to be an unstable reaction intermediate in the P5CS and P5CDH reactions. Furthermore, for simplicity in general only a single cofactor (with the highest specificity) is taken into account. #GK+GSA activity: E.C. 2.7.2.11 + E.C. 1.2.1.41.

Table S3 Free energies of formation and cellular metabolite concentrations used in the calculation of disequilibrium ratios

Metabolite	$^{\#}\Delta_f G'^0$ (kJ mol^{-1})	Reference concentration (mM)
L-Arg	210.4	†
L-Orn	160.6	†
L-Glu	-372.2	†
L-Gln	-120.4	†
L-Pro	57.97	†
D1-pyrroline-5-carboxylate (P5C)	27.96	†
α -k-Glutarate (α -k-Gla)	-633.6	†
Ammonia	82.93	1[a]
Urea	-39.74	0.2[b]
H_2O	-155.7	1.00E+03[c]
ATP	-2293	[ATP]/[ADP] = 4[d]
ADP	-1425	
Inorganic phosphate (Pi)	-1059	1[e]
NADH	1120	[NADH]/[NAD] = 0.1[f]
NAD	1059	
NADPH	237.8	[NADPH]/[NADP] = 0.5[g]
NADP	176.7	

$^{\#}\Delta_f G'^0$ the free energy of formation under standard biochemical conditions. †*Cf* Figs 2–4. [a,b]Mérigout *et al.* (2008); [c]standard biochemical condition (Alberty *et al.*, 2011); [d]estimate based on: (Gardeström & Wigge, 1988; Heineke *et al.*, 1991; Igamberdiev *et al.*, 2001); [e]estimate based on (Essigmann *et al.*, 1998; Raghothama, 1999); [f,g]estimate based on (Igamberdiev *et al.*, 2001; Shen *et al.*, 2006; Quettier *et al.*, 2008).

Table S4 Extended version of Table 2 with additional calculated values for *Poa pratensis* and *Lotus corniculatus* under ambient conditions

**Reaction**[a]	**Enzyme**[b]	$\Delta G_r^{0'}$ [c] **(kJ mol^{-1})**	$\Delta G_r^{'}$ [d] **(kJ mol^{-1})**				**[Pro]-control Median [P10;P90]**[e]			
			**Lp**	**Pp**	**Ml**	**Lc**	***Lolium perenne***	***P. pratensis***	***Medicago lupulina***	***Lotus corniculatus***
**ARG**		66.2	43	42	36	38	0.051<br>[0.023;0.11]	0.049<br>[0.022;0.11]	0.05<br>[0.023;0. 12]	0.049<br>[0.023;0. 11]
**OAT**		-26.9	-20	-22	-20	-29	0.1<br>[0.054;0.26]	0.1<br>[0.052;0.22]	0.11<br>[0.056;0.24]	0.11<br>[0.052;0.23]
**GS**		-22.8	-28	-26	-32	-29	-0.056<br>[-0.28;-0.0090]	-0.055<br>[-0.29;-0.0097]	-0.053<br>[-0.29;-0.0098]	-0.055<br>[-0.27;-0.0090]
**GOGAT**		-51.4	-44	-47	-36	-46	-0.017<br>[-0.098;-0.0016]	-0.017<br>[-0.094;-0.0020]	-0.014<br>[-0.086;-0.0013]	-0.017<br>[-0.095;-0.0017]
**GDH***		-38.1	-11	-12	-7	-14	-0.085<br>[-0.19;-0.032]	-0.081<br>[-0.19;-0.033]	-0.079<br>[-0.18;-0.033]	-0.081<br>[-0.18;-0.032]
**P5CS**		-8.33	-29	-28	-36	-34	0.056<br>[-0.0035;0.25]	0.054<br>[-0.0073;0.26]	0.039<br>[-0.013;0.24]	0.053<br>[-0.0024;0.25]
**P5CDH**		-27.8	-32	-33	-25	-27	-0.016<br>[-0.098;0.00083]	-0.016<br>[-0.11;0.0021]	-0.012<br>[-0.086;0.0039]	-0.017<br>[-0.098;0.00055]
**P5CR**		-31.1	-29	-26	-24	-22	0.069<br>[0.011;0.25]	0.07<br>[0.010;0.26]	0.064<br>[0.0059;0.25]	0.067<br>[0.011;0.26]
**PRODH**		31	25	22	20	18	-0.021<br>[-0.097;-0.0032]	-0.023<br>[-0.095;-0.0030]	-0.02<br>[-0.086;-0.0016]	-0.021<br>[-0.096;-0.0032]
	**α-k-Gla-supply**						0.77	0.7	0.71	0.7

	[0.020;2.4]	[0.080;2.3]	[0.095;2.3]	[0.060;2.2]
**Glu-supply**	0.31	0.24	0.25	0.26
	[-0.18;1.4]	[-0.22;1.3]	[-0.20;1.3]	[-0.17;1.3]
**Pro-consumption**	-0.96	-0.96	-0.97	-0.96
	[-0.99;-0.87]	[-0.99;-0.86]	[-1.0;-0.88]	[-0.99;-0.86]

[a]*Cf* Fig. 1. [b]For name *cf*. Fig. 1 and methods. [c] $\Delta G_r^{0'}$ the reaction free energy under biochemical standard conditions. [d] $\Delta G_r^{'}$ the reaction free energy adjusted for the experimental metabolite concentrations. [e]Median, tenth and ninetieth percentile values corresponding to the Pro concentration control coefficient distributions from *L. perenne* (Lp), *P. pratensis* (Pp), *M. lupulina (Ml)* and *L. corniculatus* (Lc). *The GDH reaction is taken positive in the direction of Glu production.

Table S5 Correlation analysis with correlation coefficients tabulated for the *Lolium* and *Medicago* control distributions (Figs S1 and S2, respectively)

Reaction	ARG	OAT	GS	GOGAT	GDH	P5CS	P5CDH	P5CR	PRODH	α-k-G l a	G l u	P r o-c
**ARG**	**1**	**0.64**	**-0.13**	**-0.34**	**-0.26**	**0.10**	**-0.08**	**0.19**	**-0.16**	**0.24**	**0.23**	**0.18**
		**0.63**	**-0.19**	**-0.30**	**-0.33**	**0.18**	**-0.13**	**0.17**	**-0.13**	**0.26**	**0.15**	**0.22**
**OAT**		**1**	**-0.14**	**-0.31**	**-0.23**	**0.02**	**-0.01**	**0.04**	**-0.03**	**0.26**	**0.27**	**0.04**
			**-0.14**	**-0.26**	**-0.32**	**0.05**	**-0.01**	**-0.01**	**0.03**	**0.32**	**0.25**	**-0.01**
**GS**			**1**	**0.29**	**0.15**	**-0.66**	**0.58**	**-0.51**	**0.40**	**0.13**	**0.15**	**-0.50**
				**0.35**	**0.14**	**-0.64**	**0.46**	**-0.44**	**0.40**	**0.09**	**0.22**	**-0.51**
**GOGAT**				**1**	**0.00**	**-0.30**	**0.28**	**-0.32**	**0.32**	**0.05**	**0.05**	**-0.34**
					**-0.04**	**-0.36**	**0.23**	**-0.32**	**0.26**	**0.09**	**0.08**	**-0.30**
**GDH**					**1**	**-0.09**	**0.07**	**-0.15**	**0.11**	**-0.47**	**-0.23**	**-0.12**
						**-0.03**	**0.00**	**-0.02**	**0.01**	**-0.48**	**-0.18**	**-0.09**
**P5CS**						**1**	**-0.79**	**0.60**	**-0.49**	**-0.30**	**-0.35**	**0.59**
							**-0.76**	**0.49**	**-0.43**	**-0.28**	**-0.42**	**0.56**
**P5CDH**							**1**	**-0.56**	**0.51**	**0.22**	**0.31**	**-0.63**
								**-0.47**	**0.42**	**0.21**	**0.38**	**-0.52**
**P5CR**								**1**	**-0.69**	**-0.13**	**-0.19**	**0.68**
									**-0.76**	**-0.14**	**-0.25**	**0.71**
**PRODH**									**1**	**0.09**	**0.18**	**-0.81**
										**0.16**	**0.18**	**-0.80**
**α-k-Gla-supply**										**1**	**0.27**	**-0.10**
											**0.19**	**-0.18**
**Glu-supply**											**1**	**-0.18**
**Pro-consumption**												**1**

Table S6: Comparison of changes in proline levels in plants with altered proline-biosynthesis enzymes, to changes expected on the basis of the proposed model

**Gene**	**Species**	**Manipulation**	**Pro-level Observed**	**Pro-level Predicted**	**Comp**	**Ref**
P5CS	*Arabidopsis thaliana*	KD (antisense)	↓	↓	+	Nanjo *et al*. (1999a)
P5CS	*Arabidopsis thaliana*	KO (T-DNA)	=	↓	-	Székely *et al*. (2008)
P5CS1	*Arabidopsis thaliana*	KO (T-DNA)	↓	↓	+	Sharma *et al*. (2011)
P5CS1	*Arabidopsis thaliana*	KD (allelic)	↓	↓	+	Kesari *et al*. (2012)
P5CS	*Cajanus cajan*	OE (CaMV 35S)	↑	↑	+	Surekha *et al*. (2014)
P5CS	*Glycine max*	OE (sense)	=	↑	- (+)	De Ronde *et al*. (2004)
P5CS	*Glycine max*	KD (antisense)	=	↓	- (+)	De Ronde *et al*. (2004)
P5CS	*Medicago truncatula*	OE (CaMV 35S)	↑	↑	+	Verdoy *et al*. (2006)
P5CS	*Nicotiana tabacum*	OE (CaMV 35S)	↑	↑	+	Kishor' *et al*. (1995)
P5CS	*Nicotiana tabacum*	OE (CaMV 35S)	↑	↑	+	Hong *et al*. (2000)
P5CS	*Solanum tuberosum*	OE (CaMV 35S)	↑	↑	+	Hmida-Sayari *et al*. (2005)
ProDH	*Arabidopsis thaliana*	KD (antisense)	↑	↑	+	Nanjo *et al*. (1999b)
ProDH	*Arabidopsis thaliana*	OE (CaMV 35S)	=	↓	- (+)	Mani *et al*. (2002)
ProDH	*Arabidopsis thaliana*	KD (antisense)	=	↑	- (+)	Mani *et al*. (2002)
ProDH	*Arabidopsis thaliana*	KO (T-DNA)	↑	↑	+	Sharma *et al*. (2011)
ProDH	*Nicotiana tabacum*	OE (CaMV 35S)	=	↓	-	Miller *et al*. (2009)
ProDH1	*Nicotiana tabacum*	KD (RNAi)	↑	↑	+	Ribarits *et al*. (2007)
ProDH2	*Nicotiana tabacum*	KD (RNAi)	↑	↑	+	Ribarits *et al*. (2007)
GS2	*Lotus japonicus*	KD (EMS)	↓	↑	-	D́ıaz *et al*. (2010)
OAT	*Arabidopsis thaliana*	OE (CaMV 35S)	↑	↑	+	Roosens *et al*. (2002)
P5CDH	*Arabidopsis thaliana*	KO (T-DNA)	↑	↑	+	Deuschle *et al*. (2001)
P5CDH	*Arabidopsis thaliana*	KO (T-DNA)	=	↓	-	Miller *et al*. (2009)

KD, knock-down; KO, knock-out; OE, overexpressor; Comp, compatibility. + and – indicate that observed and predicted values are match and not match under control conditions respectively; (+) indicates that observed and predicted values match when stressed plants were analysed.

Methods S1 Primers design for Q-PCR.

Insufficient genomic information is available for *L. perenne*, *P. pratensis*, *M. lupulina* or *L. corniculatus* to directly identify primers for the amplification of the gene transcripts for the enzymes for which activities were measured (i.e. P5CS, P5CR, ARG, OAT, ProDH). We therefore compared the sequences of the respective enzymes and isoforms in closely related species, and identified highly conserved regions (>95% identity) for primer design. For *L. perenne*, these species were: *Sorghum bicolor*, *Oryza sativa japonica*, *Triticum aestivum*, *Hordeum vulgare*, *Zea mays*; for *M. lupulina*, these were *M. sativa, M. trunculata, Brassica napus, B. Rapa*, *Pisum sativum*, *Arabidopsis thaliana*. At least five sequences were identified for each enzyme. P5CR, OAT, and ARG are represented by one single isoform in each of these species. For P5CS two isoforms were identified in dicots, and hence we measured both in *M. lupulina*. For ProDH, also two isoforms are identified in many species, however, only one of these is responding to stress conditions, as demonstrated in the literature (Funck *et al*., 2010).

Methods S2 Liebermeister kinetics based elasticity expressions used for metabolic control analysis.

$$\varepsilon_{Akg}^{GOGAT} = \frac{1}{1-\rho_{GOGAT}} - \frac{\alpha_{Akg}(1+\alpha_{G\ln})(1+\alpha_{NADPH})}{(1+\alpha_{akG})(1+\alpha_{G\ln})(1+\alpha_{NADPH}) + (1+\pi_{Glu})^2(1+\pi_{NADP}) - 1},$$

$$\varepsilon_{Glu}^{GOGAT} = -2\left[\frac{\rho_{GOGAT}}{1-\rho_{GOGAT}} + \frac{\pi_{Glu}(1+\pi_{Glu})(1+\pi_{NADP})}{(1+\alpha_{akG})(1+\alpha_{G\ln})(1+\alpha_{NADPH}) + (1+\pi_{Glu})^2(1+\pi_{NADP}) - 1}\right]$$

,

$$\varepsilon_{G\ln}^{GOGAT} = \frac{1}{1-\rho_{GOGAT}} - \frac{\alpha_{G\ln}(1+\alpha_{Akg})(1+\alpha_{NADPH})}{(1+\alpha_{akG})(1+\alpha_{G\ln})(1+\alpha_{NADPH}) + (1+\pi_{Glu})^2(1+\pi_{NADP}) - 1},$$

$$\varepsilon_{Orn}^{ARG} = -\left[\frac{\rho_{ARG}}{1-\rho_{ARG}} + \frac{\pi_{Orn}(1+\pi_{Urea})}{(1+\alpha_{Arg}) + (1+\pi_{Orn})(1+\pi_{Urea}) - 1}\right],$$

$$\varepsilon_{Orn}^{OAT} = \frac{1}{1-\rho_{OAT}} - \frac{\alpha_{Orn}(1+\alpha_{Akg})}{(1+\alpha_{Orn})(1+\alpha_{Akg}) + (1+\pi_{Glu})(1+\pi_{P5c}) - 1},$$

$$\varepsilon_{Akg}^{OAT} = \frac{1}{1-\rho_{OAT}} - \frac{\alpha_{Akg}(1+\alpha_{Orn})}{(1+\alpha_{Orn})(1+\alpha_{Akg}) + (1+\pi_{Glu})(1+\pi_{P5c}) - 1},$$

$$\varepsilon_{Glu}^{OAT} = -\left[\frac{\rho_{OAT}}{1-\rho_{OAT}} + \frac{\pi_{Glu}(1+\pi_{P5c})}{(1+\alpha_{Orn})(1+\alpha_{Akg}) + (1+\pi_{Glu})(1+\pi_{P5c}) - 1}\right],$$

$$\varepsilon_{P5c}^{OAT} = -\left[\frac{\rho_{OAT}}{1-\rho_{OAT}} + \frac{\pi_{P5c}(1+\pi_{Glu})}{(1+\alpha_{Orn})(1+\alpha_{Akg}) + (1+\pi_{Glu})(1+\pi_{P5c}) - 1}\right],$$

$$\varepsilon_{Glu}^{GDH} = \frac{1}{1-\rho_{GDH}} - \frac{\alpha_{Glu}(1+\alpha_{Nad})}{(1+\alpha_{Glu})(1+\alpha_{Nad}) + (1+\pi_{Akg})(1+\pi_{Nh4})(1+\pi_{Nadh}) - 1},$$

$$\varepsilon_{Akg}^{GDH} = -\left[\frac{\rho_{GDH}}{1-\rho_{GDH}} + \frac{\pi_{Akg}(1+\pi_{Nh4})(1+\pi_{Nadh})}{(1+\alpha_{Glu})(1+\alpha_{Nad}) + (1+\pi_{Akg})(1+\pi_{Nh4})(1+\pi_{Nadh}) - 1}\right],$$

$$\varepsilon_{Glu}^{GS} = \frac{1}{1-\rho_{GS}} - \frac{\alpha_{Glu}(1+\alpha_{Nh4})(1+\alpha_{Atp})}{(1+\alpha_{Glu})(1+\alpha_{Nh4})(1+\alpha_{Atp}) + (1+\pi_{G\ln})(1+\pi_{Adp})(1+\pi_{Pi}) - 1},$$

$$\varepsilon_{G\ln}^{GS} = -\left[\frac{\rho_{GS}}{1-\rho_{GS}} + \frac{\pi_{G\ln}(1+\pi_{Adp})(1+\pi_{Pi})}{(1+\alpha_{Glu})(1+\alpha_{Nh4})(1+\alpha_{Atp}) + (1+\pi_{G\ln})(1+\pi_{Adp})(1+\pi_{Pi}) - 1}\right],$$

$$\varepsilon_{Glu}^{P5CS} = \frac{1}{1-\rho_{P5CS}} - \frac{\alpha_{Glu}(1+\alpha_{Atp})(1+\alpha_{Nadph})}{(1+\alpha_{Glu})(1+\alpha_{Atp})(1+\alpha_{Nadph}) + (1+\pi_{P5c})(1+\pi_{Adp})(1+\pi_{Nadp}) - 1},$$

$$\varepsilon_{P5c}^{P5CS} = -\left[\frac{\rho_{P5CS}}{1-\rho_{P5CS}} + \frac{\pi_{P5c}(1+\pi_{Adp})(1+\pi_{Nadp})}{(1+\alpha_{Glu})(1+\alpha_{Atp})(1+\alpha_{Nadph}) + (1+\pi_{P5c})(1+\pi_{Adp})(1+\pi_{Nadp}) - 1}\right],$$

$$\varepsilon_{\Pr o}^{P5CR} = \frac{1}{1-\rho_{P5CR}} - \frac{\alpha_{\Pr o}(1+\alpha_{Nadp})}{(1+\alpha_{\Pr o})(1+\alpha_{Nadp}) + (1+\pi_{P5c})(1+\pi_{Nadph}) - 1},$$

$$\varepsilon_{P5c}^{P5CR} = -\left[\frac{\rho_{P5CR}}{1-\rho_{P5CR}} + \frac{\pi_{P5c}(1+\pi_{Nadph})}{(1+\alpha_{\Pr o})(1+\alpha_{Nadp}) + (1+\pi_{P5c})(1+\pi_{Nadph}) - 1}\right],$$

$$\varepsilon_{\Pr o}^{PRODH} = \frac{1}{1-\rho_{PRODH}} - \frac{\alpha_{\Pr o}(1+\alpha_{Nad})}{(1+\alpha_{\Pr o})(1+\alpha_{Nad})+(1+\pi_{P5c})(1+\pi_{Nadh})-1},$$

$$\varepsilon_{P5c}^{PRODH} = -\left[\frac{\rho_{PRODH}}{1-\rho_{PRODH}} + \frac{\pi_{P5c}(1+\pi_{Nadh})}{(1+\alpha_{\Pr o})(1+\alpha_{Nad})+(1+\pi_{P5c})(1+\pi_{Nadh})-1}\right],$$

$$\varepsilon_{P5c}^{P5CDH} = \frac{1}{1-\rho_{P5CDH}} - \frac{\alpha_{P5c}(1+\alpha_{Nad})}{(1+\alpha_{P5c})(1+\alpha_{Nad})+(1+\pi_{Glu})(1+\pi_{Nadh})-1},$$

$$\varepsilon_{Glu}^{P5CDH} = -\left[\frac{\rho_{P5CDH}}{1-\rho_{P5CDH}} + \frac{\pi_{Glu}(1+\pi_{Nadh})}{(1+\alpha_{P5c})(1+\alpha_{Nad})+(1+\pi_{Glu})(1+\pi_{Nadh})-1}\right].$$

Methods S3 Free energy calculations and Monte Carlo-based metabolic control analysis.

Our approach to metabolic control analysis makes use of the available structural (stoichiometric) and thermodynamic (metabolite concentrations) information. Without precise experimental knowledge about kinetic constants (such a as V_{max} or K_m) for all enzymes we use a random (Monte Carlo) sampling approach of the relative degree of saturation and the ratios of the (independent) steady state reaction rates (fluxes). The underlying assumption is that substrate and product concentrations remain within a tenfold lower to tenfold higher range of their corresponding (unknown) half saturation values (K_m).

Instead of sampling over substrate $[s]$ and $K_{m,s}$, we choose to sample over $\alpha_s = \frac{[s]}{K_{m,s}}$ from the interval [0.1;10] (and similarly over $\pi_s = \frac{[p]}{K_{m,p}}$ for product). Instead of sampling of V_{max} values (which can widely vary), through our approach we could sample the set of reaction flux ratios $\frac{J_i}{J_j}$ required for the calculation of the elasticity matrix (see below). Sampling was logarithmical within [0.1;10] intervals. In case the flux relations resulting from the stoichiometric analysis indicate that $J_i > J_j$ or $J_i < J_j$ a sampling was done over [1;10] or [0.1;1], respectively.

Monte Carlo sampling has been previously applied to control analysis (Ainscow *et al*., 1998). Besides using an easy, uniform type of Monte Carlo sampling our approach has the advantage of directly calculating control coefficients based on matrix inversion. This avoids repeated pathway simulations and perturbations for each respective parameter set. Furthermore, a generic type of enzyme kinetics has allowed us to derive elegant and broadly applicable expressions for the required elasticities (see below).

Pathway structure

	ARG	OAT	GDH	GOGAT	GS	P5CS	P5CR	ProDH	ProCo	akGPr	GluPr	P5CDH
Orn	1	-1	0	0	0	0	0	0	0	0	0	0
akG	0	-1	1	-1	0	0	0	0	0	1	0	0
Glu	0	1	-1	2	-1	-1	0	0	0	0	1	1
Gln	0	0	0	-1	1	0	0	0	0	0	0	0
P5c	0	1	0	0	0	1	1	1	0	0	0	-1
Pro	0	0	0	0	0	0	-1	-1	-1	0	0	0

The stoichiometric matrix specifies the stoichiometric numbers of the pathway variables (metabolites) relative to the various reactions (enzymes) in the columns (labelled as listed in Table S4, except ProCo: Pro consumption, α-k-GPr: α-k-Gla production, GluPr : Glu production, *cf* Fig. 7).

We defined metabolic supplies (sources) such as the ornithine pathway (Arg) and the Glu/Gln pathway (via irreversible Glu supply or 'GluPr'). *α-k-Gla*, being a metabolite involved in other major pathways was included as an extra metabolic source (irreversible supply reaction 'akGPr') and Pro consumption was added as an extra output branch (irreversible sink) to the central pathway to represent the role of Pro as substrate in for instance protein synthesis (*cf* Fig. 7; Table S4). Although the model is a simplified representation of the possible metabolic relationships, it does include the main connections to other metabolic pathways thought to play a role in Pro regulation (Verslues & Sharma, 2010). Moreover, the model was tailored to the available and relevant data, assuming that other regulatory influences can be neglected (mRNA and protein levels act implicitly only via their effect on enzyme activities). Row reduction of the stoichiometric matrix shows that the 12 modelled reactions can produce a steady state in the six independent metabolite concentrations defined as variables of the model (listed in the Materials and Methods section, *cf* Fig. 7). Six out of 12 steady state reaction rates (fluxes) are independent (reactions GS, ProDH, ProCo, α-k-Gla-Input, Glu-input, and P5CDH) and define the remaining fluxes through simple linear combinations (for instance, in the model, the flux through GOGAT is strictly equal to the flux through the GS reaction). The following relations between the reaction fluxes J_i (index *i* referring to the column number of that particular reaction) can be derived: $J_9 = J_1 + J_{10} + J_{11}$, $J_2 = J_1$, $J_5 + J_9 = J_3 + 2J_{10} + J_{11}$, $J_4 = J_5$, $J_6 = J_{10} + J_{11} + J_{12}$, $J_7 + J_8 + J_9 = 0$.

The Elasticity matrix of the model pathway (*cf* Fig. 7) is defined as follows:

$$\begin{pmatrix}
1 & 0 & 0 & 0 & 0 & 0 & -E5s1 & -E5s2 & -E5s3 & -E5s4 & -E5s5 & -E5s6 \\
0 & 1 & 0 & 0 & 0 & 0 & -E8s1 & -E8s2 & -E8s3 & -E8s4 & -E8s5 & -E8s6 \\
0 & 0 & 1 & 0 & 0 & 0 & -E9s1 & -E9s2 & -E9s3 & -E9s4 & -E9s5 & -E9s6 \\
0 & 0 & 0 & 1 & 0 & 0 & -E10s1 & -E10s2 & -E10s3 & -E10s4 & -E10s5 & -E10s6 \\
0 & 0 & 0 & 0 & 1 & 0 & -E11s1 & -E11s2 & -E11s3 & -E11s4 & -E11s5 & -E11s6 \\
0 & 0 & 0 & 0 & 0 & 1 & -E12s1 & -E12s2 & -E12s3 & -E12s4 & -E12s5 & -E12s6 \\
0 & 0 & \frac{J9}{J1} & -\frac{J10}{J1} & -\frac{J11}{J1} & 0 & -E1s1 & -E1s2 & -E1s3 & -E1s4 & -E1s5 & -E1s6 \\
0 & 0 & \frac{J9}{J2} & -\frac{J10}{J2} & -\frac{J11}{J2} & 0 & -E2s1 & -E2s2 & -E2s3 & -E2s4 & -E2s5 & -E2s6 \\
\frac{J5}{J3} & 0 & \frac{J9}{J3} & -\frac{2\,J10}{J3} & -\frac{J11}{J3} & 0 & -E3s1 & -E3s2 & -E3s3 & -E3s4 & -E3s5 & -E3s6 \\
\frac{J5}{J4} & 0 & 0 & 0 & 0 & 0 & -E4s1 & -E4s2 & -E4s3 & -E4s4 & -E4s5 & -E4s6 \\
0 & 0 & 0 & \frac{J10}{J6} & \frac{J11}{J6} & \frac{J12}{J6} & -E6s1 & -E6s2 & -E6s3 & -E6s4 & -E6s5 & -E6s6 \\
0 & -\frac{J8}{J7} & -\frac{J9}{J7} & 0 & 0 & 0 & -E7s1 & -E7s2 & -E7s3 & -E7s4 & -E7s5 & -E7s6
\end{pmatrix}$$

with the relations between the steady state fluxes through the various reactions represented by the J_i ratios. *i* refers to the reaction corresponding with the *i*-th column of the stoichiometric matrix.

The entry $E_i s_j$ represents the elasticity coefficient $\varepsilon^{v_i}_{[s_j]}$ of reaction *i* with regards to changes in metabolite s_j. For instance:

$$\varepsilon^{OAT}_{[Orn]} = \frac{\partial \ln v_{OAT}}{\partial \ln [Orn]} = \frac{[Orn]\partial v_{OAT}}{v_{OAT}\partial [Orn]} .$$

For a reaction with *i* substrates and *j* products, assuming no allosteric regulation the Liebermeister rate law is as follows:

$$v = V_f \frac{\prod_i \alpha_i^{h_i n_i} \left(1 - \frac{\Gamma'}{K_{eq}'}\right)}{\prod_i (1+\alpha_i)^{h_i n_i} + \prod_j (1+\pi_j)^{h_j n_j} - 1}, \qquad \text{(Eqn 1)}$$

with V_f the V_{max} of the forward reaction, α_i the concentration of enzyme substrate *i* relative to the K_m value for that metabolite, π_j the concentration of enzyme product *j* relative to the K_m value for that product, h_i the cooperativity or Hill index of species *i* (set to 1 here), n_i the stoichiometric coefficient of species *i*, Γ' the mass-action ratio under biochemical reference conditions, and correspondingly K_{eq}' the equilibrium constant.

Pathway thermodynamics

Molar reaction free energies under reference conditions were calculated ($\Delta_r G^{'0}$, Tables 2, S1; *cf* the Materials and Methods section). We have used the $\Delta_r G^{'0}$'s to calculate the $\Delta_r G^{'}$'s which specify the direction of the thermodynamically spontaneous reaction and hence in the context of reversible enzyme kinetics the direction of the net (positive) flux.

The physiological standard state of pressure *p* = 1 atm, temperature *T* = 298.15, *pH* 7 was used, with an ionic strength *I* of 0.25 M (Alberty, 2003). For most biochemical compounds involved in the pathway reactions (Table S3) free energies of formation $\Delta_f G^{'0}$ in those conditions were available from Alberty (2003). However, for proline, arginine, and ornithine, only estimates of the free energy of formation $\Delta_f G^0$ at the chemical standard condition of *pH* 0 and zero ionic strength are reported (http://opencobra.sourceforge.net/openCOBRA/Welcome.html; Jankowski *et al.*, 2008). In that case the following formula was used to calculate $\Delta_f G^{'0}$ (in accordance with equation 4.4-10 of Alberty (2003):

$$\Delta_f G^{'0} = \Delta_f G^0 (I = 0) + N_H RT \ln(10) pH - \frac{2.91482\left(z^2 - N_H\right) I^{0.5}}{1 + 1.6 I^{0.5}}$$

with *I* the ionic strength, N_H and *z* the change in the number of hydrogen atoms and charge, respectively, *R* the universal gas constant and *T* the absolute temperature.

The fractions of the various ionization states of the polyprotic acids at *pH* 7 were calculated based on reported pK_a values of the respective functional groups and used to determine the N_H and *z* values. The role of temperature differences with the lab experiments are expected to be relatively small based on test calculations with the van ‘t Hoff equation and were therefore neglected. Since no values could be found for the 1-pyrroline-5-carboxylate we used the ‘group contribution’ method (Jankowski *et al.*, 2008) to estimate the $\Delta_f G^0$ value and calculate the $\Delta_f G^{'0}$ as described. All $\Delta_f G^{'0}$ values (Table S2) were then combined to calculate the reaction free energies and finally equilibrium constants according to:

$\Delta_r G^{'0} = \sum_{i=1}^{N} v_i \Delta_f G^{'0} = -RT \ln K_{eq}^{'}$, with v_i the stoichiometric coefficient of species *i*.

The reaction free energy at concentrations (activities) different from 1 M (except for water) was calculated as follows:

$$\Delta_r G^{'} = RT \ln \frac{\Gamma^{'}}{K_{eq}^{'}}$$

with Γ' the mass action ratio.

Since the thermodynamic state of the pathway does not differ much between species and conditions only the calculated values for *L. perenne* (*Lp*) and *M. Lupulina* (*Ml*) in normal climate conditions are reported. $\Delta G_r^{'}$'s for ARG are in conflict with its expected role in hydrolysing Arg as part of the urea cycle (Verslues & Sharma, 2010), indicating that the reaction's mass-action ratio has been potentially overestimated. One possible cause could be our estimate of the urea concentration (0.2 mM: based on Mérigout *et al.*, 2008). A second possible cause is that this part of the proposed pathway predominantly takes place in the mitochondria (Verslues & Sharma, 2010) making the estimates based on total cell extracts not representative. The same applies to the ProDH reaction, which is expected to proceed in the direction of Pro breakdown. Since its $\Delta_r G^{'0}$ (and mass-action ratio) is near the values obtained for the related reaction of P5CR, this reaction in principle should be spontaneous in the anabolic direction. Assuming a higher mitochondrial than cytoplasmic NAD/NADH ratio can resolve this issue. Therefore, the derived disequilibrium ratios were arbitrary adjusted for ARG (to 0.5, i.e. close to equilibrium) and for ProDH (to 0.1, i.e. moderately far from equilibrium).

References

Ainscow EK, Brand MD. 1998. Errors associated with metabolic control analysis. Application Of Monte-Carlo simulation of experimental data**.** *Journal of Theoretical Biology* **194:** 223–233**.**

Alberty RA. 2003. Standard transformed Gibbs energies of coenzyme A derivatives as functions of pH and ionic strength. *Biophysical Chemistry* **104:** 327–334.

Alberty RA, Cornish-Bowden A, Goldberg RN, Hammes GG, Tipton K, Westerhoff HV. 2011. Recommendations for terminology and databases for biochemical thermodynamics. *Biophysical Chemistry* **155:** 89–103.

De Ronde J, Cress W, Krüger G, Strasser R, Van Staden J. 2004. Photosynthetic response of transgenic soybean plants, containing an *Arabidopsis P5CR* gene, during heat and drought stress. *Journal of Plant Physiology* **161:** 1211–1224.

Deuschle K, Funck D, Hellmann H, Daeschner K, Binder S, Frommer, WB. 2001. A nuclear gene encoding mitochondrial Delta-pyrroline-5-carboxylate dehydrogenase and its potential role in protection from proline toxicity. *Plant Journal* **27:** 345–356.

Díaz P, Betti M, Sánchez DH, Udvardi MK, Monza J, Márquez AJ. 2010. Deficiency in plastidic glutamine synthetase alters proline metabolism and transcriptomic response in *Lotus japonicus* under drought stress**.** *New Phytologist* **188:** 1001–1013.

Essigmann B, Güler S, Narang RA, Linke D, Benning C. 1998. Phosphate availability affects the thylakoid lipid composition and the expression of *SQD1,* a gene required for sulfolipid biosynthesis in *Arabidopsis thaliana***.** *Proceedings of the National Academy of Sciences, USA* **95:** 1950–1955.

Funck D, Eckard S, Müller G. 2010. Non-redundant functions of two proline dehydrogenase isoforms in Arabidopsis. *BMC Plant Biology* **10:** 70.

Gardeström P, Wigge B. 1988. Influence of photorespiration on ATP/ADP ratios in the chloroplasts, mitochondria, and cytosol, studied by rapid fractionation of barley (*Hordeum vulgare*) protoplasts. *Plant physiology* **88:** 69–76.

Heineke D, Riens B, Grosse H, Hoferichter P, Peter U, Flügge U-I, Heldt HW. 1991. Redox transfer across the inner chloroplast envelope membrane. *Plant Physiology* **95:** 1131–1137.

Hmida-Sayari A, Gargouri-Bouzid R, Bidani A, Jaoua L, Savouré A, Jaoua S. 2005. Overexpression of Δ1-pyrroline-5-carboxylate synthetase increases

proline production and confers salt tolerance in transgenic potato plants. *Plant Science* **169:** 746–752.

Hong Z, Lakkineni K, Zhang Z, Verma DPS. 2000. Removal of feedback inhibition of Δ1-pyrroline-5-carboxylate synthetase results in increased proline accumulation and protection of plants from osmotic stress. *Plant Physiology* **122:** 1129–1136.

Igamberdiev AU, Bykova NV, Lea PJ, Gardeström P. 2001. The role of photorespiration in redox and energy balance of photosynthetic plant cells: a study with a barley mutant deficient in glycine decarboxylase. *Physiologia Plantarum* **111:** 427–438.

Jankowski MD, Henry CS, Broadbelt LJ, Hatzimanikatis V. 2008. Group contribution method for thermodynamic analysis of complex metabolic networks. *Biophysical Journal* **95:** 1487–1499.

Kesari R, Lasky J, Villamor JG, Des Marais D, Chen Y-J C, Liu TW, Lin W, Juenger T, Verslues P. 2012. Intron mediated alternative splicing of *Arabidopsis P5CS1* and its association with natural variation in proline and climate adaptation. *Proceedings of the National Academy of Sciences, USA* **109:** 9197–9202.

Kishor PK, Hong Z, Miao G-H, Hu C-AA, Verma DPS. 1995. Overexpression of [delta]-pyrroline-5-carboxylate synthetase increases proline production and confers osmotolerance in transgenic plants. *Plant Physiology* **108:** 1387–1394**.**

Mani S, Van de Cotte B, Van Montagu M, Verbruggen N. 2002. Altered levels of proline dehydrogenase cause hypersensitivity to proline and its analogs in *Arabidopsis*. *Plant Physiology* **128:** 73–83.

Mérigout P, Lelandais M, Bitton F, Renou J-P, Briand X, Meyer C, Daniel-Vedele F. 2008. Physiological and transcriptomic aspects of urea uptake and assimilation in *Arabidopsis* plants. *Plant Physiology* **147:** 1225–1238.

Miller G, Honig A, Stein H, Suzuki N, Mittler R, Zilberstein A. 2009. Unraveling delta1-pyrroline-5-carboxylate-proline cycle in plants by uncoupled expression of proline oxidation enzymes. *Journal of Biological Chemistry* **284:** 26482–26492.

Nanjo T, Kobayashi M, Yoshiba Y, Kakubari Y, Yamaguchi-Shinozaki K, Shinozaki K. 1999a. Antisense suppression of proline degradation improves

tolerance to freezing and salinity in Arabidopsis thaliana. *FEBS Letters* **461:** 205–210.

Nanjo T, Kobayashi M, Yoshiba Y, Sanada Y, Wada K, Tsukaya H, Kakubari Y, Yamaguchi-Shinozaki K, Shinozaki K. 1999b. Biological functions of proline in morphogenesis and osmo-tolerance revealed in antisense transgenic *Arabidopsis thaliana*. *Plant Journal* **18:** 185–193.

Quettier A-L, Shaw E, Eastmond PJ. 2008. *SUGAR-DEPENDENT6* encodes a mitochondrial flavin adenine dinucleotide-dependent glycerol-3-P dehydrogenase, which is required for glycerol catabolism and postgerminative seedling growth in Arabidopsis. *Plant Physiology* **148:** 519–528.

Raghothama K. 1999. Phosphate acquisition. *Annual Review of Plant Physiology and Plant Molecular Biology* **50:** 665–693.

Ribarits A, Abdullaev A, Tashpulatov A, Richter A, Heberle-Bors E. 2007. Two tobacco proline dehydrogenases are differentially regulated and play a role in early plant development. *Planta* **225:** 1313–1324.

Roosens NH, Al Bitar F, Loenders K, Angenon G, Jacobs M. 2002. Overexpression of ornithine-δ-aminotransferase increases proline biosynthesis and confers osmotolerance in transgenic plants. *Molecular Breeding* **9:** 73–80.

Sharma S, Villamor JG, Verslues PE. 2011. Essential role of tissue-specific proline synthesis and catabolism in growth and redox balance at low water potential. *Plant Physiology* **157:** 292–304.

Shen W, Wei Y, Dauk M, Tan Y, Taylor DC, Selvaraj G, Zou J. 2006. Involvement of a glycerol-3-phosphate dehydrogenase in modulating the $NADH/NAD^+$ ratio provides evidence of a mitochondrial glycerol-3-phosphate shuttle in *Arabidopsis*. *Plant Cell* **18:** 422–441.

Surekha C, Kumari KN, Aruna LV, Suneetha G, Arundhati A and Kishor PK. 2014. Expression of the *Vigna aconitifolia P5CSF129A* gene in transgenic pigeonpea enhances proline accumulation and salt tolerance**.** *Plant Cell, Tissue and Organ Culture* **116:** 27–36.

Székely G, Abrahám E, Cséplo A, Rigó G, Zsigmond L, Csiszár J, Ayaydin F, Strizhov N, Jásik J, Schmelzer E *et al*. 2008. Duplicated *P5CS* genes of Arabidopsis play distinct roles in stress regulation and developmental control of proline biosynthesis. *Plant Journal* **53:** 11–28.

Verdoy D, De La Pena TC, Redondo FJ, Lucas MM, Pueyo JJ. 2006. Transgenic *Medicago truncatula* plants that accumulate proline display nitrogen-fixing activity with enhanced tolerance to osmotic stress**.** *Plant, Cell & Environment* **29:** 1913–1923**.**

Verslues PE, Sharma S. 2010. Proline metabolism and its implications for plant–environment interaction. *The Arabidopsis Book/American Society of Plant Biologists* **8:** e0140
